# A Treatise on Food and Diet, with Observations on the Dietetical Regimen Suited for Disordered States of the Digestive Organs; and an Account of the Dietaries of Some of the Principal Metropolitan and Other Establishments for Paupers, Lunatics, Criminals, Children, the Sick, &c.

**Published:** 1844-01

**Authors:** 


### BRITISH AND FOREIGN
### MEDICAL REVIEW,

FOR JANUARY, 1844.

### PART FIRST.

### Analytical and Critical Reviews.

### Art. I.

1. *A Treatise on Food and Diet, with Observations on the Dietetical Regimen suited for Disordered States of the Digestive Organs; and an Account of the Dietaries of some of the principal Metropolitan and other Establishments for Paupers, Lunatics, Criminals, Children, the Sick, &c.* By Jonathan Pereira, M.D. F.R.S. & L.S.—*London*, 1843. 8vo, pp. 542.

2. *A Treatise on Diet, comprising the Natural History, Properties, Composition, Adulterations, and Uses of the Vegetables, Animals, Fishes, &c. used as Food.* By William Davidson, M.D. M.R.C.S.E.—*London*, 1843. 12mo, pp. 383.

3. *Food, and its Influence on Health and Disease, or an Account of the Effects of different kinds of Aliment on the Human Body. With Dietetic Rules for the Preservation of the Health.* By Matthew Truman, M.D.—*London*, 1842. 12mo, pp. 240.

The works placed at the head of this article, taken collectively, profess to comprise the entire subject of diet. While examining them, we shall endeavour to ascertain the progress that has been made in the attainment of correct principles of dietetics; for it may be affirmed that such principles are still a great desideratum in medical practice. As a collateral object, we have it in view to illustrate the importance to mankind of every step gained in this inquiry, not only in a physical and hygienic, but in an intellectual, moral, and religious point of view.

Dr. Pereira's treatise is unquestionably the earliest attempt that has been made, in this country at least, to place the general subject of dietetics upon a strictly scientific basis. Accordingly, in the present article we shall follow his arrangement, and avail ourselves of many of his valuable suggestions as we proceed. Chemistry in its present state, and especially the organic chemistry of Prout and Liebig, is made by far more available in elucidating the question of diet by this physician than by any writer who has preceded him. He abandons the ordinary method of arranging foods according to the proximate principle which happens to predominate in their composition, and adopts one of a more scientific character, partly from Tiedemann, and partly original. Under the term "Food" Dr. Pereira comprises not only solids, to which the word "Aliment" has frequently been restricted, but also Fluids and Condiments. The work is divided into two parts, the first treating of "the substances employed by man as food;" the second of "the adaptation of aliment to the different wants and conditions of human existence."

### I. OF FOODS.

This part is divided into three chapters, and comprises an account of the chemical elements of food; of alimentary principles, as oil, sugar, protein or fibrin, which are compounds of two, three, four, or more undecompounded bodies; and of compound aliments, as the flesh of animals, vegetables, wheat flour, &c., which are frequently composed of many alimentary principles: each of these divisions requires a special exposition.

I. ELEMENTS OF FOOD. This chapter contains numerous chemical details of the highest importance, introduced by Dr. Pereira for the first time into a systematic treatise on diet. They are chiefly derived from the recent productions of Boussingault, Liebig, Payen, and Dumas. Physiologists variously estimate the number of elements which enter into the human body as essential constituents; with Dr. Prout, the author assumes it to be thirteen. These are all derived from the food.

1. *Carbon.* This is an essential constituent of every living tissue, and it ultimately serves as fuel for combustion, and the production of animal heat. More or less appears to be required by the system, not so much for the purposes of nutrition, but, according to the varying circumstances of the production of heat, in states of exertion or rest for instance; or according to external temperature as determined by season, climate, the state of the atmosphere, and we may add clothing. It is derived from the food, as a component part of the different alimentary principles, in proportions varying from about 34 to 78 per cent., the smaller proportions being contained in certain vegetables, and the largest quantity in oils and fats. Dr. Pereira shows by calculation that the chemical changes effected upon the carbon received into the system are nearly, if not quite, sufficient to explain the animal temperature.

2. *Hydrogen.* This also, as an element of the organized tissues, is an essential constituent of food. Where it occurs in the relative proportion to oxygen, which is necessary to constitute water, as in starch and sugar, the substances are regarded as hydrates of carbon, and they yield the latter element only, to combine with oxygen in the system. For this reason the quantity of oxygen inspired by graminivorous animals, is equivalent to the carbonic acid expired, no oxygen being required for the combustion of hydrogen. When the hydrogen is in excess, as in fat, alcohol, protein, and gelatin, whatever intermediate forms the food may assume in the animal solids or fluids, not only is its carbon converted by combustion into carbonic acid, but its excess of hydrogen is converted into water. So that in carnivorous animals, which consume a large quantity of super-hydrogenous food, a larger quantity of oxygen is inspired than of carbonic acid expired, this being an additional source of animal heat. When the hydrogen is deficient in an alimentary substance, as in citric and tartaric acid, all the hydrogen and part of the carbon being already in combination with oxygen, none of the first element, and probably a part only of the second, can be available as fuel for animal combustion.

3. *Oxygen.* This element is a constituent of the more essential parts of the vital tissues, and accordingly of food. In the latter it bears a proportion varying from 10 to 140 parts, combined with 120 parts of carbon. Those foods—as fat and proteinized substances—which contain a small proportion of oxygen, must require a larger quantity of respired air for their combustion, than those—as sugar and starch—which contain a larger proportion of oxygen. The latter produce less heat than the former. Hence the quality of the food as respects its elements, and the activity of respiration, reciprocally influence each other; theory in this instance confirming and explaining the results of experience. Oxygen has an interest of the highest importance, distinct from that of food. As vital air it is the supporter of combustion, and of that degree of heat without which no vital action can continue; its ultimate effects being at the same time destructive, consuming the greater number of the products of vital action; but it does not appear that the oxygen of the food is employed in the system for these purposes. The consumption of atmospheric oxygen, and the heat produced, is greater from a diet of animal principles than from an equal quantity of vegetable matter; it is increased by the use of spirituous liquors, the alcohol of which contains no oxygen.

4. *Nitrogen.* This is also an essential constituent of every vital tissue. Many of the most important alimentary substances are destitute of it; hence the distinction of food, so much insisted upon by Liebig, into nitrogenized, as fibrin and albumen, and non-nitrogenized, as sugar and starch; and it being held that the former only can nourish the vital tissues, while the latter are wholly or in part burned; the former have been called "plastic elements of nutrition," and the latter "elements of respiration." Nitrogen is a constituent of the food of the embryo chick and of the young mammal. The various nitrogenized substances in vegetable as well as animal food contain from 14 to 18 per cent., or proportions nearly identical with those of the tissues which they serve to nourish. Food in its complicated state contains from about 1·3, as in rice, to 15 or 18 per cent. as in albumen or gelatin. It has been assumed that the quantity of nitrogen is a measure of the nutritive value of food, and Dr. Pereira gives Boussingault's scale of nutritive equivalents constructed upon this principle.

5. *Phosphorus* is a component part of bones, nerves, muscles, and other parts of the living body, and of course a necessary element of the food of animals. It is a constituent of the yelk of eggs and of milk, and of the greater part of the animal food employed by man. It is also abundant in vegetable food, in the forms of phosphates of ammonia, lime, or magnesia. More phosphorus is supplied to the body under ordinary diet than its wants require, the excess being eliminated in the urine or solid excrements; but some vegetable aliments, as beans and peas, are remarkably deficient in this element.

6. *Sulphur* is again an essential constituent of various tissues, as of fibrine, hair, and bones, and must be received into the system with the food. The nitrogenized alimentary principles of vegetables, also eggs and milk, water (in the form of sulphate of lime), and numerous compound aliments, contain it. It is eliminated from the system in the urine as sulphates, partly formed by the action of the oxygen of arterial blood on the sulphur of the metamorphosed tissues.

7. *Iron.* This element is found invariably in the blood-corpuscles, although according to Scherer it is neither essential to hematosin nor necessary to the colour of the blood. Its quantity is about two parts in the thousand, varying in individuals of different temperaments and in different states of health. It is a constituent also of hair. Many articles of food contain iron, as yelk of egg, milk, mustard, potatoes, peas, &c.; but having been found in the gastric juice of the dog and in the chyle, Dr. Pereira regards this as an explanation of how it gets into the blood. Excepting the hair, no account is given of an outlet or organ of waste for this metal.

8. *Chlorine* is a constituent of blood, and of various secretions and excretions as a compound of sodium; and of the gastric juice as a compound of hydrogen. It is indispensable to health, and is constantly passing out of the system with the secretions, hence it requires to be constantly renewed, and forms an essential part of food in every period of life, being a constituent of milk and both of the yelk and white of egg.

9. *Sodium* is an essential constituent of blood, of the solids, of the secretions, and especially of bile. It is furnished by most animal foods, but not by all the vegetables usually employed;—is for the most part taken with the food as a condiment, and received into the system in the form of hydro-chlorate of soda, passing out of it again by the urine in the same form, and also in flesh-eating animals, as sulphate and phosphate of soda.

10. *Calcium.* This is a component part of all animals, and particularly of their shells, crusts, and bones; also of most of the solids, and of blood, occurring in the form of subphosphate of lime. It is introduced with the food as a constituent of white and yelk of egg, of cereal grains and onions as a subphosphate, of rhuburb as an oxalate, of grapes as a tartrate, &c. Gum and unrefined sugar also yield calcium. Most animal foods contain it, and the water we drink contains its sulphate and bicarbonate. Yet the system appears occasionally to suffer from a deficient supply of calcium.

11. *Magnesium* occurs, combined with oxygen and phosphoric acid, and often with ammonia, in minute quantities in various animal structures, as blood, teeth, bones, and nervous matter. It is a constituent of both animal and vegetable food, as of cereal grains, potatoes, eggs, and flesh.

12. *Potassium.* A similar statement may be made of this element; it is required for the blood, and for the formation both of the solids and secretions, being a component part of most plants employed as food which grow inland. Of grapes and potatoes for instance. It is also contained in minute portions in common salt.

13. *Fluorine.* It may be remarked here that the statement by Berzelius, that fluorine is a constituent of bones and teeth is confirmed by Marchand. Dr. G. Owen Rees affirms, on the contrary, with Fourcroy and Vauquelin, that no such substance exists in the animal body. It has never been detected in plants, and the source of it, as an element of food, is unknown.

The above enumeration of the elements of matter required for the nutrition of the human body, must be taken conventionally, for it is well known that others have been detected. Copper in milk, sweat, and in the intestines, and manganese constantly in the hair, as also in biliary and urinary concretions, and in the opaque lens, for instance. The account given of their origin is tolerably satisfactory, and were it not that chemical research has developed facts not easily explained, on the assumption that food is the sole source of the material fabric, it would be quite so. The first remark that suggests itself, on carefully considering this part of the subject, refers to the question, whether the vital force is capable of forming and decomposing what are regarded as the elements of matter. Dr. Prout threw out a hint, as early as 1822, that such a circumstance may happen, from considering the quantity of earthy matter contained in the skeleton of the chick. More recently he has stated his belief in the possession of this power by living systems under certain extraordinary circumstances. But with all deference to so high an authority, it is difficult to think otherwise than that, if calcium be actually formed in the chick, it must be in accordance with some general law of nature, and not an extraordinary or incidental event. If azote or carbon be decomposed by the vital force, such decomposition must occur constantly to answer certain ends in the animal economy. All the recent discoveries in animal chemistry tend to explain the origin ab externo, not only of the elements but even of many of the compounds detected in the animal tissues, and no new fact has been developed to confirm Dr. Prout's notion. Dr. Pereira is accordingly warranted in assuming that the calcium found in the skeleton of the chick is derived from one or more of the constituent parts of the egg, and that the elements detected by analysis in the various parts and products of animals have an external origin.

Another point, of even greater importance than the former, is the question whether these elements are received into the system, to become a part of the vital structures, otherwise than as aliment, and by the digestive organs. Liebig is a high authority on the negative side of this question, which has been most discussed in reference to the origin of the nitrogen. In this country there appears to be a strong opinion among practical chemists and physiologists, that, at all events when the food with which animals are fed, is deficient in nitrogen, this element becomes absorbed in the lungs. Dr. Prout suggests also, in the last edition of his work, that atmospheric air involved during mastication is a source of azote to vegetable feeders, and contributes to the formation of azotized compounds in the alimentary canal. (p. 504.) The principal arguments employed by Liebig, in favour of his view of the case, are briefly these: nitrogen is not created in the system, nor have we any satisfactory evidence that it is absorbed from the atmosphere in the vital process; the food of all animals contains nitrogenized compounds, identical in composition with the principal constituents of the blood and organized tissues, and therefore, substances devoid of this element are not required for the production of these constituents; the quantity of the nitrogenized principles of food consumed is amply sufficient for the nutrition and waste of the body; and the non-nitrogenized foods alone are incapable of supporting animal life. On the other side, Dr. Pereira advances a long argument, of which the following are the principal items. Many physiologists believe, from the results of experiments, that nitrogen is absorbed in the lungs: we have proof that non-nitrogenized compounds really do take part in the transformations of the animal tissues, and of the possibility of their conversion into the nitrogenized constituents of an animal body, in the case of benzoic acid appearing as hippuric acid in the urine: in animals that feed on vegetable food, Dr. Prout detected no albumen in the chyme until after it had passed the pylorus, and sugar is not found in the blood, from which it may be inferred that in the duodenum, the saccharine is converted into the albuminous principle: diet even of fibrin, or of any nitrogenized principle exclusively, is incapable of supporting life. Dr. Pereira further remarks, that if nitrogenized aliment is derived from plants ready formed, the fact that fibrin, albumen, and gelatin, taken together or separately, are incapable of supporting animal life, while gluten from wheat or maize is alone sufficient to satisfy complete and prolonged nutrition, as stated by the commissioners of the French academy in 1842, remains unexplained. Neither is the necessity very obvious of more complex organs of digestion for the herbivora than for the carnivora. A hint is also given that nitrogen may be derived from the ammonia of the atmosphere, but this is unsupported by any proof except the analogy of plants, which, according to Liebig, assimilate nitrogen from that source.

We cannot undertake to settle this important question; but the remark here suggests itself, that so long as it remains undetermined, one class of physiologists believing in the formation of fibrin, for instance, from the nitrogen of the atmosphere, another class denying that nitrogen gas is ever assimilated, and affirming that nitrogenized compounds are received into the system only in a state of ready formation, it will be impossible to settle certain points in reference to food and diet. If Liebig's opinion is not established conclusively to every mind, neither is it invalidated by Dr. Pereira's reasoning. This can only be done by experiments on a wider scale. On some points there has certainly been a misapprehension of Liebig's meaning. Thus, by the expression "no nitrogen is absorbed from the atmosphere," taken with the context, this chemist manifestly means that no nitrogen from the atmosphere is employed in forming the azotized compounds of the blood, nor in what Dr. Prout terms secondary assimilation. It may also be here remarked, that protein, the basis of these compounds, is newly discovered, and its qualities by no means completely ascertained. This base, or complex radicle, has to be searched for in the chymous mass, and it is highly desirable that some of our practical chemists should inform us in what state it exists in the "incipient albumen," met with in the alimentary canal, as described by Dr. Prout.

Although there are other channels by which the elements of the human body may be received into the system, as during the absorption of water by the skin, there is perhaps, after all, little room to doubt that, under ordinary circumstances, they are mainly if not altogether derived from the ingesta. Dr. Pereira has done a good service in making the chemical elements of food a distinct consideration in dietetics, and in future all treatises on this branch of science must be considered imperfect in which it is omitted. Already is it manifest that health depends upon a due and proportionate supply of these elements, considered apart from the organic combinations into which they are formed, and that an excess or deficiency can no longer be separated from our notions of the causation of disease. Facts before us sanction the opinion, that regarding them in their elementary point of view, the quantities and proportions in which they are supplied to individuals, races, and generations, are, with other external agencies, intimately concerned in the production of temperaments and varieties of constitution. Truly our knowledge under this head is meagre, but we are in possession of a few strong facts. The satisfactory results of experiments made to determine the quantity of iron in the blood, and the effects which follow an addition or subtraction of this element, are in point. Nor can it be doubted, were our investigations pushed far enough, that equally conclusive evidence would be obtained as respects calcium, potassium, sulphur, &c. At the same time it is equally true that these elements require to be supplied to the animal system in certain forms of combination. We cannot depend upon phosphorus, or sulphur, or any other substance being assimilated to organic principles by the vital force when exhibited in the simple state; although it is highly probable that this may occur. When employed, even as medicines, the form of combination is of the utmost importance, as illustrated in the very obvious effects of certain preparations of the metal just mentioned. This element is assimilated by the blood much more rapidly when the citrate than when the sulphate is employed, a fact which may be explained by the powerful affinities exerted by the acid of the latter. When the citrate becomes absorbed the organic acid is destroyed by combustion, and the iron is readily yielded up, to enter into the structure of the blood-corpuscle. Thus, as between citrate and sulphate of iron, so, in the matter of diet, the supply of any particular element rarely admits of being regarded as a simple proposition. As in the case of nitrogen, extremely useful scales of the relative value of foods, measured by their elementary constitution, may be constructed upon the principle of Boussingault's scale of equivalents, before alluded to, yet these scales can only be regarded as approximations to the real or comparative nutritive qualities of the various articles of food.

II. Alimentary Principles. Proceeding to examine the second chapter of the work before us, we find, in the food of animals, that the elements to be employed in the economy are combined together in certain groups of two, three, or more, constituting distinct substances, the properties of which have been separately studied. To distinguish the nutritive from the innutritive parts of our aliment is no new doctrine. Hippocrates was aware that particular parts of food only are destined to take the nature and form of the human body. Many of the older authors regarded the nutritive principle as being always uniform, foods differing from each other in the proportions they contain. Others held that alimentary matters of whatever nature, must be converted into this uniform nutritive principle. Boerhaave, regarding all animal food as originally derived from vegetables, recognized aqueous, saponaceous, oily, and spirituous principles, as separable from the grosser materials; and Cullen reduced the alimentary matter of vegetables to three substances, acid, sugar, and oil. Dr. Fordyce classed vegetable foods accordingly as they contained sugar, farinaceous matter, oil, gum, and several indefinite substances called mucilage; the nutritive principles of animal foods being referred to coagulable mucilage, lymph, and oil; believing that all the animal solids are composed of mucilage and water. It is, however, to Dr. Prout that we are indebted for the first rational account of the alimentary principles, who referred them to four great classes, the aqueous, the saccharine, the albuminous, and the oleaginous. Dr. Pereira, for reasons in some instances adduced, to which we shall refer, has extended these to twelve classes. As with the elements, we propose to describe them seriatim, reserving our principal commentary till afterwards.

1. *Water* constitutes three fourths of the weight of the human body. It is less immediately essential to our existence than air, and more so than solid food, being probably the only natural drink after the period of infancy. Many of the purposes fulfilled by water are well known. It is furnished to the system both by aqueous drinks and by many of the solid substances employed as food. The latter contain very variable proportions: thus, wheat-flour, 14·5, fresh meat about 75, cow's milk about 87, and beef-tea 98·4 per cent. These are instances taken promiscuously from a very useful table, at p. 80, of the proportions of water in different articles of diet. Whether water ever yields up its elements to assist in the formation of the organic tissues of animals is uncertain, but, according to Liebig, the hydrogen of vegetable tissues is derived from it. Dr. Prout also appears to admit that such a power is occasionally exerted by animals, and either water or its elements are intimately concerned, as shown both by Prout and Liebig, in the metamorphoses which other alimentary principles undergo in the living system. Its importance as a solvent, both in primary and secondary assimilation, and in the removal of the effete materials of the body, is equally well established. Many of the processes essential to animal life depend upon the chemical action of water, as the formation of the hydrochloric acid of the gastric juice, and of the soda of bile, from the chloride of sodium of the blood. It may be added that the quantity of water employed by the system amounts to several pounds daily, varying according to external temperature, exercise and rest, the quantity and quality of the materials received into the digestive organs or into the blood, and other causes; and while water is essential to the process of digestion, it is also rapidly absorbed by the blood-vessels of the alimentary canal, and conveyed through the portal system, holding many substances in solution, respecting which our information is at present very defective.

2. *The mucilaginous principle.* Numerous vegetable substances employed as food contain gum, in proportions varying from 1, as in rice, to 19·37 per cent., as in the kidney-bean, or even more; its constituents are carbon and the elements of water, and it is regarded by Liebig as an element of respiration. Experience shows that in its isolated state this substance is by no means easily digested in the human stomach.

3. *The saccharine principle.* This is also composed of carbon and of the elements of water in somewhat variable proportions. Respecting the use of this principle in the economy, the greatest difference of opinion prevails. Dr. Prout regards it as a nutritious substance, and the type of one class of his alimentary principles, and indeed of vegetable aliments generally; holding, that during digestion and secondary assimilation it is convertible into the oleaginous and albuminous principles, and into blood. Liebig admits that it may be formed into fat, but denies that it is ever converted into azotized compounds, or employed in the nutrition of the azotized tissues; he classes it with gum as an element of respiration, its carbon being employed as fuel for the production of animal heat.

4. *The amylaceous principle.* This is one of the most abundant constituents of vegetable food, occurring in quantities which vary from 3 to 72 per cent. Sago, tapioca, arrowroot, cassava, tous-les-mois, potato-starch, salep, rice-starch, &c., are here treated of separately as articles of diet, consisting almost entirely of starch. This principle is regarded in the same point of view as sugar, both by Prout and Liebig, except that it forms a necessary part of human food,—may be employed in much larger quantities than sugar, and its use persisted in for an unlimited period. According to Liebig, when in excess, under favorable circumstances, it contributes to the formation of fat, but is incapable of transformation into the azotized materials of blood. Both sugar and starch are easily digested in the human stomach.

5. *The ligneous principle.* This is introduced rather in deference to an opinion of Dr. Prout than from a conviction in Dr. Pereira's mind, that it is capable of yielding nutriment to man. It is true that it forms the appropriate food of various insects, and of some of the lower animals, and when reduced by various processes, it is said to form a substance analogous to the amylaceous principle. But many of the facts relating to the use of bark and wood as food, by the Laplanders and other tribes, may be accounted for by the formation and the diffusion of starch through every part of the plant, in the autumnal sap. So that starch may in reality be the nutritive principle of bread made of bark and wood. The ligneous matter of our ordinary vegetable foods is evacuated with the fæces, as indigestible refuse, being sometimes of use as a mechanical stimulus to the bowels, as the bran of wheat; and sometimes injurious, as the husks of fruit.

6. *The pectinaceous principle.* Vegetable jelly. This includes pectine and pectic acid, one or both being contained in most pulpy fruits, as currants, apples, oranges, and in many vegetables, as artichokes, carrots, and turnips. Unripe fruits contain but a very small quantity. The dietetical properties of vegetable jelly are very little known, but it is readily digested, and is probably slightly nutritive. Its components are carbon and the elements of water, plus oxygen, and like other principles of this nature, it is classed by Liebig as an element of respiration. Dr. Pereira remarks, that in consequence of the excess of oxygen in relation to hydrogen, the use of vegetable jelly may be to diminish the function of respiration. It is uncertain whether the tendency which fruits have to promote alvine evacuations depends upon this principle.

7. *The acidulous principle.* Dr. Pereira's reasons for admitting this, are, that vegetable acids constitute one of the most constant ingredients of our foods; that fruits and succulent herbs have been in use both in ancient and modern times, and vinegar from a very early period; and that the employment of vegetable acid appears, from various statements before us of the effects which result from complete and prolonged abstinence from vegetables and fruits, or their preserved juices, to be necessary for the preservation of health. The pure acids will not in every way supply the place of the natural juices, and all vegetable juices are not equally beneficial. Some of the acids, as the oxalic, tartaric, and gallic, form combinations with bases before they enter the circulation, and these compounds appear in the form of salts in the urine. Others, as the tartaric, malic, and acetic, suffer decomposition in their transit; their carbon being converted into carbonic acid, and their salts appearing as carbonates in the urine. It will be observed that the principles already described comprise the saccharine group of Dr. Prout.

8. *The alcoholic principle.* This belongs to the oleaginous group of Prout. According to Liebig it is another element of respiration. Dr. Pereira, adopting the latter view, regards it, under some circumstances, as an alimentary principle. Its carbon and hydrogen act as fuel in the animal economy, and in cases of extreme suffering and exhaustion its "cautious and moderate dietetical use" has, on many occasions, proved invaluable. The use made of alcohol by coachmen to avoid "catching cold," and Captain Bligh's account of the good effects of a little rum served out in teaspoonfuls, in lessening the sufferings of himself and companions, arising out of the mutiny of the crew of the Bounty, are cited as examples.

9. *The oily principle.* Vegetable and animal foods contain fixed oils and fats composed of the well-known fatty and saponifiable principles. According to Liebig, these oily principles, composed chiefly of carbon and hydrogen, are incapable of forming the essential parts of blood; and, like other non-nitrogenized principles, they serve as fuel for the support of respiration. Dr. Prout holds, on the other hand, that, like sugar, they are convertible into most, if not all, of the matters necessary for the existence of animal bodies. But oily diet alone is incapable of supporting life; animals fed upon it exclusively soon die of inanition. The oily globules from the food appear to be conveyed into the blood, and to be deposited in the adipose tissue, or they contribute to the formation of the nervous tissues. The principal aliments of this class are fat, suet, marrow, butter, and olive and other vegetable oils.

Volatile oils form the material of flavour and odour of numerous productions of the vegetable kingdom employed at table either as aliment or as condiments; as of thyme, parsley, onions, &c. The oils are for the most part absorbed, and subsequently eliminated, without losing their characteristic qualities; or they may in part be burnt, and produce heat. "They stimulate but do not seem to nourish;" their principal effect being that of gratifying the palate.

10. *The proteinaceous principle.* This group differs from the albuminous alimentary class of Dr. Prout, in the omission of gelatinous substances. The dietetic properties of pure protein have not been ascertained, but its compounds form the plastic elements of nutrition of the German school. Protein is formed only in vegetables, the animal organism possessing the power of converting its various modifications into each other. Hence the importance of vegetables to animals as the exclusive source of the most essential material of their nutriment. The fibrin and albumen of blood, the substance of every variety of muscle, and of all the important viscera, the casein of milk, and accordingly the nutriment of the young, are equally dependent upon a supply of this principle. The organic material of the brain and nerves, being distinct in composition from that of all other animal tissues, is formed in the animal body only, and its formation takes place "from compounds of protein, either by the loss of some azotized compound, or by the addition of carbonized products, as fat." The proteinaceous food employed by man is derived both from animals and vegetables. In his infancy from the caseum of milk, and in after life from the muscles, viscera, and fluids of animals; and from the seeds and other parts of plants. *Animal fibrin* is contained in variable quantities in the more usual articles of animal diet; for instance, in the muscle of beef, together with albumen, there is 20 per cent., in the sole 15 per cent., and in sweetbread 20 per cent. Fibrin speedily dissolves in the living stomach, and even in dyspeptics is regarded as of easy digestion: but, employed exclusively, it is incapable of supporting life. *Animal albumen* constitutes perhaps the most important part of animal foods. The white of eggs contains 15 per cent. Like the former substance, it is of easy digestion and highly nutritious. The gastric juice dissolves it, coagulating it in the first place, if liquid. Liebig regards it as the true starting-point of the animal tissues; with the aid of air, of the oily matter of the yelk, and of iron, the whole being derived from it during the incubation of the chick. Yet animals cannot subsist on albumen alone, but refuse it and die of starvation when it constitutes their sole diet. *Animal casein* is distinguished from albumen and fibrin by its great solubility. As it occurs in milk it is of easy digestion. Liebig asserts that no foreign substance is required to convert it into blood, and that it contains a much larger portion of the earth of bones in a very soluble form, than blood does; indicating how completely dependent the development of the vital organs in animals is upon the supply of a substance identical in organic composition with the chief constituents of blood. Milk contains from 1·52 to 4·50 per cent. As it occurs in cheese, this principle is of much more difficult digestion, requiring for its solution in the stomach as much as three and a half hours. The vegetable proteinaceous substances are also *fibrin, albumen,* and *casein;* the analyses of which do not differ from each other, or from those of the corresponding animal principles, more than two analyses differ of one and the same substance. Their nutritive powers are equal to those of the proteinized substances derived from animals. The quantities of these principles in vegetables are extremely variable; peas and beans, for instance, contain 14 or 15 per cent. of legumine or casein; rice, from 3 to 4 per cent. of fibrin or gluten; the dried juice of the carrot a little more than 4 per cent. of albumen; the potato ·5 per cent. of albumen, and ·055 per cent. of gluten. If proteinized principles be deficient in the food of herbivorous animals, nutrition is arrested; and carnivora in consuming the flesh and blood of herbivora consume in fact only the vegetable principles which have served for the nutrition of the latter. As Fordyce described it, before chemistry had determined the composition, even of water or air—"The lion may live on the horse, but the horse derives its nourishment from grass, and those animals which live on the flesh of such other animals as are sustained by vegetables may be considered as ultimately living on vegetable food."

18. *The gelatinous principle.* Dr. Prout regards gelatin as a modification of albumen, or, as "the least perfect kind of albuminous matter existing in animal bodies." This substance, as an article of food, is derived from isinglass, and from the skins, tendons, and bones of animals, and also from cellular membrane; differing considerably in its properties and its digestibility, as obtained from different sources. Gelatin differs from albumen in its chemical properties and composition, and for this and other reasons its nutritive uses cannot be identical with those of the proteinized compounds. Liebig considers that gelatin is formed in the animal body from some of the compounds of protein, but, at the same time, that gelatinous food may contribute directly to the nourishment of the gelatinous tissues. Animals die of starvation if attempted to be nourished on gelatin alone. M. Donné tried its effects on himself; "at the expiration of six days he had lost two pounds' weight, and during the whole time was tormented with hunger, and suffered from extreme faintness, which was only alleviated after dining in the usual way." Still, the daily experience of the physician proves that gelatin, in conjunction with other alimentary substances, assists in nutrition.

12. *The saline principle.* Saline matters being essential constituents of the blood and of the organized tissues and secretions, they are necessarily component parts of our food, and among the most important, in a dietetical point of view, as entering most largely into the living solids and fluids, are common salt and the earthy phosphates. To these may be added the salts of potassa and the compounds of iron.

Although chloride of sodium is a constituent of many articles of food and of drink, yet these do not contain a sufficient quantity to supply the wants of the system ; as a component of the blood, an excess or deficiency of this salt appears to affect the constitution of the blood-corpuscle, and from the salt of the blood the gastric juice derives the chlorine of its hydrochloric acid, and the bile obtains the sodium of its soda ; the addition of salt to our food is therefore a necessary practice. The earthy phosphates are constantly present in the ashes of animal substances, and being necessary constituents of these, as well as of bones, they must be supplied to the system, and are accordingly component parts both of animal and vegetable food. Corn, potatoes, milk, eggs, and the flesh and blood of animals furnish, in fact, more than the demand, the excess being eliminated in the secretions. The salts of potassa are also derived both from animal and vegetable food, and all the nutritive plants contain compounds of iron, which element reappears in the blood and flesh of animals.

Our examination of the chapter of which the above is an abstract, induces us to suggest the question—what is an alimentary principle? It is manifestly not simply a proximate principle of organic substances, according to the meaning attached to that term by the chemists; although many of these are truly alimentary principles. Quinia is a proximate, but not an alimentary principle. Dr. Prout, who first made a specific application of the term, grouped several proximate materials together, to constitute one great alimentary principle. This is exemplified in the albuminous and the saccharine groups. Moreover, he holds that the diet of man, and of the higher classes of animals, to be complete, must contain more or less of all his "staminal principles;" this being illustrated in the case of milk, the only substance prepared by nature expressly for food, which contains water, a saccharine, an albuminous, and an oily matter; and again, in the food of the more perfect animals, composed, for the most part, of substances referrible to three if not to four of these staminal groups. So far, this may be considered a philosophical account of the essential constituents of food. But there is another point of view in which these principles may be regarded, viz., as identical in composition, and in the arrangement of their elements, with the materials that compose the structures they are intended to produce or to nourish. Here Dr. Prout's arrangement fails, for there is no tissue in the animal system of which the saccharine principle is the prototype, and there are tissues which have no prototype in the food, as the nervous. The progress of discovery recently, has rendered this identity in the essential constituents of food, compared with those of the animal structures, much clearer; it has furnished new instances, and, in the school of Liebig, it has gone so far as to render it doubtful, whether the living tissues of the body are ever nourished otherwise than by the elements of which they are composed being introduced from without, in forms of combination more or less nearly allied to their own. If this doctrine were well established, there would be no question about the true basis of the science of dietetics. Dr. Pereira's "alimentary principles," however, answer neither the one nor the other of these descriptions. They appear to be compounds introduced into the digestive organs as aliment, and capable of being appropriated to any necessary, or even useful purpose, in the economy—a totally different affair; and although we are not disposed to quarrel with a name, the wisdom of the designation given to them, viewed as a whole, may be fairly doubted.

Dr. Pereira makes gum an alimentary principle distinct from sugar; not that both are essential to the nutrition of the body, but, as we read it, because gum may be employed as a dietetical and probably as a nutrient agent. Most complete have been the revolutions of opinion respecting this substance. For many years it was regarded as the most nutritious part of vegetable substances, and by some, as the only nourishment they afford; an opinion, supported by a fact, noticed by Fordyce, and quoted from him by many succeeding writers, that men have been nourished by "gum seneca" and water for many weeks, in caravans which had lost their way in the sandy deserts of Africa. Magendie's experiments, proving that animals fed on gum alone soon die of inanition, led to the totally opposite opinion, that it has no nutritive powers; and indeed it has been looked upon by some as serving no useful purpose in the economy. Liebig describes it, after passing the digestive organs, as fuel for combustion. Prout assigns to it the capability of being converted, within the economy, into some more essential nutritive compound, but of this we have no experimental proof. With respect to the story told by Hasselquist, the traveller above referred to, any opinion founded upon the use of a substance such as gum, in its crude state, and under the circumstances described, must be inconclusive. Gum senegal, in particular, is well known to differ from gum arabic, having a slightly bitter flavour. It has been said that an Arab can live upon six ounces of gum daily, but he takes it dissolved in milk, and recent experiments appear to show that gum arabic contains a small portion of azote.

Lignine is introduced upon the bare suspicion of its being a distinct principle capable of yielding nutriment to man. Pectine and pectic acid, again, have no very distinct nutritive properties. Even those materials of the ingesta, which appear to serve no other purpose in the economy but as fuel for combustion and the production of animal heat, taking no part in the nutrition of the vital structures, as taught by Liebig and his disciples, are still, while following Liebig's doctrine, separately regarded as alimentary principles; alcohol, for instance. On the other hand, some data are given, to render it probable, that the acidulous principle serves unknown important purposes; the introduction of certain salts into the blood may be suggested. The facts published by Dr. Baly of the occurrence of scurvy in public institutions, where the diet is defective in vegetable acids, and the immediate cessation of that disease when vegetables containing organic acids are given, as potatoes, seem to confirm this opinion. The propriety of separating the gelatinous from the proteinaceous principle is obvious; and as to the saline substances, there is as much reason to regard the chloride of sodium, and probably phosphate of lime, as also iron in some of its combinations, in the light of distinct alimentary principles, as any other combination of the elements of matter employed as food.

The reasoning employed in this volume does not convince us that alcohol in any of its forms of mixture, ought to be regarded as an alimentary principle. This class of substances should surely be limited, at least, to combinations of the elements of matter, which are necessary or wholesome to be presented to the digestive organs, (for the purposes whether of nutrition or respiration,) in the physiological or healthy state of the animal economy; and under ordinary circumstances. Admitting the facts cited by Dr. Pereira, they prove at most, in our minds, that alcoholic compounds, when judiciously resorted to, are excellent remedial agents, and may be employed with advantage to counteract the effects of certain abnormal states into which the constitution of man is liable to be precipitated; be it by his follies or by virtue of his enterprising nature. Seeing no reason to doubt the rationale of its operation, adopted from Liebig; or that it serves to warm the system, when this is exposed to undue causes of depression of temperature; or to excite the organic functions, when they have become excessively depressed; such effects must be held to be therapeutical rather than dietetical; and although, in consequence of the very general employment of alcohol by civilized man, it is important to treat of it in a work on food and on precepts of diet, it cannot be proper to rank it as an approved constituent of ordinary wholesome alimentation.

The controversy at present raging as to the source of the fat of animals, whether introduced from without, or formed within the system, has an important bearing on the general subject of assimilation, primary and secondary. Liebig, comparing the quantity of fat deposited with the quantity of the oleaginous principle contained in the food, came to the conclusion that fat must be formed in the body by the abstraction of oxygen from starch, sugar, and other non-azotized food. Dumas, Payen, and Boussingault afterwards proved that the food employed in the experiment, contained much more oily matter than Liebig was aware of—that maize, instead of containing very little, has from four to nine per cent.; rendering it in some degree probable that all the fat of the animal was derived ready formed from the food. There are many most interesting facts in favour of Liebig's view; thus, bees being fed with honey alone, quite free from wax, one part of wax was produced for every twenty parts of honey consumed. The analogy between wax and oil, and the near identity of honey and sugar, seem to indicate that oily and waxy substances are formed in the economy from food. In a recent analysis, Dr. G. O. Rees found a large quantity of oil in the chyle of a donkey, first kept for many hours without food and then fed with beans; but beans are said to contain no oil, and the lymph of the animal at the same time furnished a much smaller proportion of oil than the chyle.

Since this controversy commenced, Peleuze appears to have discovered that on submitting sugar to a slow fermentation, under circumstances as nearly as possible analogous to animal digestion, the formation of butyric acid, that is to say, of one of the constituents of animal fats, takes place. This is one of the questions, upon the settlement of which our knowledge of the real digestive powers of the higher orders of animals in a great measure depends. Are Liebig and Dumas both right?—the fatty matter being usually derived from that which preexists in the food, and the system having the power of elaborating it from saccharine compounds when the supply from the former source is deficient.

A most striking circumstance connected with these alimentary principles—that no one of them, in its isolated state, is capable of affording nourishment to animals for any length of time, calls for remark in this place. They all excite aversion after they have been used for a very short time. Gluten is the only compound, belonging in anywise to this class of substances, which has been found to be consistent with prolonged nutrition. The glutinous part of wheat was first discovered early in the last century by Beccaria, who, with Rouelle, proved that to it wheaten flour and some other vegetable substances owe their superiority as human food. According to a table given by Dr. Pereira, the proportion of glutinous matter contained in different varieties of wheat, varies from 9·2 to 35·1 per cent. Now this "Beccaria's gluten" is a mixture of several organic principles. By boiling in alcohol it may be resolved into an insoluble portion, being vegetable fibrin, and a soluble part, consisting probably of at least two substances, *mucin* and *glutin;* and, as remarked by Magendie, this substance, containing much gluten (v. fibrin), "combined with a little albumen, gum, mucilage, fecula, and even sugar," is really a very compound substance. Hence its sufficiency for the nourishment of animals. A consideration of these facts leads to the conclusion, that protein and its compounds undergo some important changes in the digestive organs, to prepare them for secondary assimilation, and that they require to be mixed or combined with other substances in order to ensure the due progression of these changes.

The above remarks are in no way intended as a censure of Dr. Pereira's labours. They are made rather in exemplification of the imperfect state of dietetics as a science, and in anticipation of those improvements which may be hoped for in the third part of the organic chemistry, promised to the public by Professor Liebig.

III. Compound Aliments. The third and last chapter of the first part of the work before us treats of the different varieties of food as it is furnished by the markets for our tables. These consist of animal and vegetable substances in their prepared or unprepared state, every individual of which contains two or more, and sometimes many, of the alimentary principles already described. Food has been regarded, in this point of view more especially, in preceding treatises on the subject, and Dr. Pereira's work differs from others, in the chemical and dietetical properties of the alimentary principles as well as of the elements being made essential parts of this branch of medical study. We shall here have to notice Dr. Davidson's and also Dr. Truman's productions, although both the one and the other must be looked upon rather as intended for the general than the scientific or medical reader. They will be found, however, to contain numerous interesting facts, and in some respects they supply what may possibly be regarded as omissions in the one at the head of our list, which we have chosen as a text.

That the particular kinds of food employed by different nations, and in various climates and regions of the earth, is the result of accident, necessity, and conventional usage, rather than a selection made upon rational principles, must be sufficiently obvious. In fact, no such thing as principles in dietetics could be known or surmised by man in his primitive state, and very few have been discovered in the various stages of his advance towards civilization. Still, as in other branches of hygiene and medicine, experience—although very frequently deceptive—has for the most part proved herself a faithful guide, and we deem it advantageous to be made acquainted with the customs of different nations as respects the sustenance of their bodies. In these times also, when food for the multitude is too certainly a pressing want, it behoves us to consider all possible sources thereof, and the dietetical value of every available natural production. "Quæ in terris gignuntur omnia ad usum hominum creantur," and as animals exercise their instinct in obtaining food, so man must bring his reason to determine the problem of providing aliment for multiplying generations.

We find it noticed by Dr. Truman that horse-flesh is not an uncommon article of diet in Denmark and Sweden, and a large quantity is consumed as food in Paris. The Tartars, Arabs, and Patagonians eat asses. Dogs were formerly much employed as food in Europe; they are also eaten by the Chinese, and by some savage nations, and even in civilized Paris;—evidence is in fact adduced that dog's flesh is sweet and wholesome meat. Elephants are considered delicacies in Cochin China; camel's flesh in Egypt; and in China, again, rats and moles are sold by weight in the markets, for food. Whales, walruses, seals, bears, beavers, otters, badgers, foxes, &c., are eaten by the inhabitants on the coasts of the polar seas; and the Caffres consume lions. So also, many birds and their eggs, which are foreign to our notions as articles of diet, are employed as such in different countries; and the same may be affirmed of crocodiles and various reptiles and serpents; of white ants, caterpillars, centipedes, snails, earthworms, and even human flesh. Among vegetables, chestnuts are used as a wholesome food in Lombardy by the lower classes. In Norway, during seasons of scarcity, the peasants eat chaff and the inner bark of pine trees, which are ground and baked to render them digestible. The woody fibre also of beech, birch, elm, lime, and poplar, will serve for diet when properly prepared. Acorns are said to be the principal vegetable food in California; and some nations find nourishment in earth. On the other hand, that the proscription of the productions of nature as food, by different nations, is frequently the result of prejudice, is exemplified in the facts collected by Dr. Truman.

"The inhabitants of several immense tracts of the globe entertain a marked distaste for milk; the Chinese, the inhabitants of Java, and of the other islands in the Indian Archipelago, have almost as great an aversion to it as we should have to blood; and similar objections extend also to cheese and butter among these people." (p. 29.)

Pork is eschewed by Mohammedans, Jews, and Copts, in whatever clime they live; and the ox by various castes of Hindoos,—by religious ordinances; and these articles of ordinary diet with us are, no doubt, from education and long prejudice, by them disliked. Several tribes of people in Abyssinia have a perfect abhorrence of the flesh of the common fowl, and whole nations have rejected the use of fish.

Without professing to give credit to every account of cannibalism and geophagism recorded by travellers; or, in this place, to explain under what circumstances, for what length of time, and with what additions, chaff and earth can afford sustenance to mankind, it is quite clear that innumerable natural productions, not ordinarily so regarded, may be appropriately employed as food. The knowledge we have obtained of their constituent and alimentary principles teaches us in a great measure how, as a matter of course, it must be so, and reconciles numerous apparent inconsistencies. Extensive strata in various parts of the earth consist almost entirely of infusory animalcules, as shown by Ehrenberg; and, in particular, the "mountain meal" employed in Lapland, in 1832, with the bark of the birch tree and a little flour, for making bread, was discovered by Retzius to contain nineteen species. At present, our appreciation of the relative digestibility and of the nutritive value of these substances is very limited; but it is satisfactory to observe the investigation of these points in progress as a branch of science. In the mean time, not only must the remark quoted by Dr. Truman from Herschel be generally concurred in—that some of the facts before us "deserve a higher degree of celebrity than they have obtained, because they prove that absolute famine may be rendered next to impossible,"—but the question must force itself upon the reflecting mind; how is it possible, in a civilized community, that want and starvation should at any time be stalking abroad? Yet such things are.

From the time of Pliny, the natural history of the animals and vegetables from which food is derived, has been a subject of considerable interest with the medical practitioner; and the works of Tournefort and the Jussieus sufficiently established, from an early period, that the organic characters, the principles, and the virtues of plants are closely allied. The study of comparative anatomy and physiology has also its value. Dr. Pereira, on account of the extent of the subject, has purposely excluded all natural historical details. Dr. Davidson, on the contrary, professes to give them; in fact, they form a considerable part of his book. Nutriment may be distinguished from poison by an examination of the structure and analogies, and of the simple qualities of natural productions. We are willing to admit also that a knowledge of the native climate of our domestic animals, of their habits, propensities, modes of forming their nests, plumages, loves, &c., is all very proper for a medical man to be acquainted with; and Dr. Davidson's work will be found interesting in this way; but we do not quite understand that the pugnacity of lobsters, the manner in which fish is caught, or the method of storing away potatoes belongs, properly, to a 'Treatise on Diet,' by a grave physician.

Dr. Pereira arranges compound aliments under three primary divisions. 1, Solid foods, or aliments proper; 2, Liquid foods, or drinks; 3, Seasoning agents or condiments. This arrangement is not carried out very logically, for while broths and soups are classed as liquids, milk is placed amongst solids; it is, however, convenient, and the best, perhaps, in the present state of our knowledge, that could be devised. The first division has two sections; 1, Animal foods; 2, Vegetable foods.

1. *Animal Foods.* These are treated of in the order of the zoological classes, the author confining himself to animals employed as food in this country. Belonging to mammalia are the ox, sheep, hog, deer, rabbit, and hare. Of which it may be affirmed that the blood, the milk, many other fluids, and every structure, are called into requisition as alimentary substances. Bones, for instance, of which those of the ox and sheep are chiefly used in making soup, afford gelatin. But soup prepared from bones has higher nutritive powers than gelatin in its isolated state,—a circumstance to be accounted for upon a principle before adverted to,—complexity of composition; for bones yield phosphate and carbonate of lime, magnesia, soda, potassa, and also fat. Again, muscular flesh, or ordinary "butcher's-meat," must not be regarded as fibrin only, but as composed of tendon, aponeurosis, fascia, nerve, vessel, cellular tissue, blood, sinew, and fat; and as yielding to the organs of primary digestion,—aqueous, saline, fatty, gelatinous, and proteinaceous alimentary principles. Of birds, a very large number are employed as food; the more important being the common fowl, the pheasant, partridge, pigeon, duck, and goose, of which the flesh, viscera, and eggs abound in albumen, fibrin, gelatin, and oil. The only reptile introduced is the green or edible turtle; the part eaten being rich in gelatin, and poor in fibrin. From the class of fishes, man obtains an endless variety of food, some nations, both in ancient and modern times, deriving from it their chief sustenance. The parts employed are the gelatinous integument, the flesh, and the viscera. The flesh is watery, and composed of fibrin, albumen, and gelatin, sometimes mixed with oil, as in the salmon and the eel, the latter principle being more abundant in the thinner or abdominal parts than in the thicker or dorsal portions; hence the preference given by epicures to the thinnest part of salmon. The white curdy matter, observed between the flakes of boiled fresh fish, is a film of albumen, produced by the coagulation of the serous juices intervening between the muscular layers. Of the crustacea many are edible, having a white firm flesh, which contains much gelatin, as in the lobster, crawfish, crab, and shrimp. Lastly, of the mollusca, a few species only are used in this country; the oyster, mussel, and periwinkle may be mentioned as examples. The flesh of the oyster, according to the analysis of Pasquier, contains fibrin, albumen, gelatin, osmazome, mucus, water, and salts.

The composition and qualities of the particular articles of animal diet vary exceedingly. Dr. Pereira has collected a few analyses, but it must be confessed that this part of organic chemistry has not at present been very completely followed out; we can only illustrate some of its results in their application to dietetics. It has already been shown that animal foods exhibit considerable differences in the quantity and proportions of their elements, and of those more essential organic compounds which have been called alimentary principles, and, accordingly, in their nutritive powers. Water is the largest constituent. Dr. Pereira remarks that, "as 100 parts of the flesh of the oyster contain only 12·6 parts of solid matter, while 100 parts of butcher's meat contain, on an average, about

25 parts, it is obvious that oysters must be less nutritive than butcher's meat." Of the proteinaceous principle, the milk of different animals contains from 1·52 to 4·50 per cent. Of the gelatinous principle, or matter which may be transformed into gelatin, bones contain from about 17 to 27 per cent., and fish, flesh, and fowl from 5 to 7 per cent. The oleaginous principle almost entirely constitutes animal fats; the various kinds of milk contain from ·11 to 4·20 per cent. The yelk of egg 28·75 per cent. Of the saline principles, which are very numerous in animal food, we have no accurate data, in fact their proportions are frequently too small to admit of being determined by our present methods of analysis. The elementary constitution of animal as compared with vegetable food, is much more closely allied to that of the blood and solids, for the nourishment of which it is intended. The ultimate composition of flesh is identical with that of blood, and while the alimentary principles occur in a much more concentrated state, than for the most part they do in vegetables, the different kinds of animal food exhibit among themselves less variety of constitution.

2. *Vegetable foods* are arranged after Tiedemann, partly by the method of natural history, and partly by the organs employed. There are two primary classes: I, Aliments derived from flowering plants; II, Aliments derived from flowerless plants. The first class has seven orders according to the parts used as food,—seeds, fruits, leaves, &c. The orders of the second class are botanical, viz., ferns, lichens, algæ, and fungi.

The present chapter contains such a vast mass of detail, including numerous chemical analyses, that we can only undertake to give a general view of it, with an occasional allusion to principles available in dietetics. The first class of vegetables is by far the most important. *Order* A,—comprises the seeds, among which the cereal grains contain an abundance of the vegetable nitrogenized materials, identical in composition with those of animal bodies, but diluted—so to speak—largely, with non-nitrogenized constituents. The proximate principles of these grains are starch; vegetable albumen; v. fibrin, gluten, and mucin, and oily matter, constituting raw or ordinary gluten; also, sugar; gum; earthy phosphates; ligneous matter in the form of bran, husks, &c.; and water. But of these, the different species and the varieties of each species of grain furnish very different proportions. Taking protein and its compounds as a standard, wheat is the most nutritious; oats, according to Boussingault's analysis, are nearly equivalent, then come rye and barley. Rice contains only from 3 to 4 per cent. of any proteinized principle, and above 80 per cent. of starch;—and maize, another most important source of human sustenance, appears to contain 5 or 6 per cent. of the former, to about 80 of the latter, with a considerable quantity of oil, and some gum and sugar. The various kinds of bread in general use by mankind are made of these cereal grains. Next in importance are the leguminous seeds, of which, chiefly, peas and beans are used in this country, containing from 10 to 14 or 15 per cent. of vegetable casein, a little gum and albumen, but no oil. The oily seeds, as the almond, walnut, &c., are composed of a large proportion of fixed oil; the former, according to Boullay's analysis, of 54 per cent. Thus the seeds of plants furnish an abundance of all the staminal principles of Dr. Prout; they were among the most primitive materials employed, and still constitute the largest portion of the food of mankind. Their greatest deficiency is in the organic acids.

*Ord.* B. Of the fleshy fruits of the earth, a vast variety are eaten, and many are important articles of diet. None contain more than the most minute quantity of nitrogenized matter. Water forms the greater part of all, but its proportion lessens, and the quantity of solid matter increases as they become ripe; being in the drupaceous fruits from 70 to 90 per cent. Cucurbitaceous fruits are the most watery, the cucumber containing 97·14, and the melon 98·5 per cent. Gum, pectin, sugar, and the acids also enter into their constitution. When ripe, the quantity of sugar is in general greatly augmented:—in the cherry, for instance, from 1·11 to 18·12 per cent. The acids are chiefly the citric, tartaric, and malic; occurring free, or in combination with bases. Fruits contain also volatile oils, colouring matters, lignin, and particular extractives; and each fruit has in general some peculiarity in its composition, upon which its sensible and some of its dietetical qualities depend. Dates constitute a considerable portion of the food of many of the inhabitants of Egypt, Arabia, and Persia; according to a recent analysis by Reinsch, they are composed of more than half sugar, with pectin, pectinaceous gum, bassorin, fatty oil, and wax, but no nitrogenized principle; so that the chief nutritive material would appear to be sugar. The fig, which is eaten so much as food in eastern countries, also contains 62·5 per cent. of granular sugar. The olive, grape, oranges, lemons, pineapple, mulberries, strawberries, raspberries, and many others, are separately treated of;—space will not admit of any detail respecting them.

*Order* C consists of roots, subterraneous stems, and tubers; including the turnip, carrot, parsnip, beet, potato, and Jerusalem artichoke. The turnip contains 92·5 parts of water to 7·5 parts of solid matter, and a minute quantity of two nitrogenized substances,—v. albumen, and v. fibrin; but the most important substance in this order is, undoubtedly, the potato; its constituents being water, starchy matter, ligneous matter, v. fibrin, v. albumen, and gluten, oil, gum, asparagin, extractive, vegetable acids, salts, and occasionally, solanina. According to Einhoff, its acid is the tartaric combined with potassa and lime, but by Vauquelin it is regarded as the citric,—partly free and partly in combination with those bases: in its raw state it contains from about 66 to 75 per cent. of water.

The remaining orders include:—*Or.* D, buds and young shoots; as the onion tribe and asparagus, the former containing an acid vegetable oil, and the latter asparagin, a peculiar nitrogenized compound, regarded by Liebig as a nutritive agent:—*Or.* E, leaves and leafstalks; as the the cabbage, cauliflower, brocoli, watercress, lettuce, rhubarb, turnip-tops, spinage, &c., the green colour depending upon chlorophyle, a substance in the form of globules, in nature between resin and fat, and but little acted on by the digestive organs:—*Or.* F, receptacles and bracts; the artichoke, which contains a saccharine and mucilaginous juice, with starchy matter, being the only individual mentioned:—*Or.* G, stems; furnishing farinaceous substances, as sago.

The second class of vegetables comprises—a few tuberous roots or rhizomes, used only in times of scarcity;—certain lichens, which owe their alimentary qualities to a starchy matter usually accompanied with a bitter principle,—hence they require to be prepared before they are employed for food. Some are eaten by the hunters in the arctic regions under the name of "Tripe de Roche." Also certain species of algæ, which abound in mucilaginous or vegeto-gelatinous substances, to which they owe their dietetical uses; they also contain starch, and sometimes sugar. Carrageen, or Irish moss, is an example, the carrageenin being more nearly allied to pectin than to any other vegetable principle. Of the last order—fungi—three species only are used as food in this country, while in Russia they employ thirty-three species. The supposed alimentary principle of mushrooms is fungin, a substance allied to lignin, but, according to Braconnot, highly nitrogenized, although Vauquelin obtained from it little nitrogen, and Müller considers fungin to be one of the more simple nutritive substances.

3. *Liquid aliments or drinks.* The compounded liquid aliments are arranged in six orders. *Or.* A, *Mucilaginous, farinaceous, or saccharine drinks;* called "slops" in this country, and "tisans" on the continent. They differ but little from common water, have slight nutritive powers depending upon gum, sugar, &c., held in solution, and are employed as diluents and demulcents. *Or.* B, *Aromatic or astringent drinks.* This includes tea, coffee, chicory, chocolate, and cocoa. Tea contains 12 to 18 per cent. of tannic acid, also volatile oil, gum, albumen, resin, &c., and theine, (c. 8, h. 5, n. 2, o. 2,) a salifiable base, existing in combination with a part of the tannic acid. Hot water extracts the tannate of theine and also the tannin, but in cooling both substances precipitate. Theine, with the elements of water, and an excess of oxygen, is equivalent in composition to taurine. Liebig has accordingly suggested that tea may have some direct influence over the formation of bile; and that in sedentary persons, who take little azotized food, and in whom there is little waste of matter, tea may benefit the health by furnishing materials to form bile. The flavour of tea and its influence on the nervous system depend on its volatile oil. Coffee contains theine and several peculiar principles, as caffeic acid, upon which, probably, its odour depends. Like tea, it diminishes the disposition to sleep. The infusion or decoction of chicory forms a perfectly wholesome beverage. Chocolate kernels, or cocoa seeds, differ exceedingly from the last-mentioned substances, they contain 53·10 per cent. of oil, and 16·7 per cent. of albumen, with starch, mucilage, and lignin; also theobromine, (c. 9, h. 5, n. 3, o. 2,) which, from its analogy to theine, Liebig suggests, on hypothetical grounds, may contribute to the formation of bile and urine. Cocoa is somewhat less oily than chocolate, and rather astringent; when properly made it is freed from much of its oil.

*Or.* C, *Acidulous drinks.* They consist of water as a basis, and, usually, of a vegetable acid; being prepared with the juice of fruits, or by dissolving acids or acidulous salts in water, or by the decoction of fruits; as lemonade, imperial, and apple tea, respectively. Both the acid and water contribute to allay thirst. In this order are arranged carbonated or effervescent drinks, as soda water, &c. *Or.* D, *Drinks containing gelatin and osmazome.* These are essentially decoctions of meat, but frequently contain vegetables. By the agency of water and heat in making broths and soups—bones, aponeuroses, cellular tissue and tendons yield gelatin; fatty matters are melted and diffused; and various products are obtained by unknown reactions.—*a.* Creatine, a nitrogenized crystallizable substance. *b.* Osmazome, the extractive upon which the odour and flavour chiefly depend. *c.* Ammonia. *d.* A sulphuretted compound. *e.* A volatile acid, analogous to acetic acid. *f.* An odorous volatile acid, similar to butyric acid. The last three being wholly or partially volatilized. The vegetables employed communicate colouring, mucilaginous, saccharine, azotized, and sometimes sulphuretted matters, with volatile oils and salts. The amount of nutritive matter in broth does not exceed 3·5 per cent. Beef-tea, mutton, veal, and chicken broths, are the lightest forms of animal food. *Or.* E, *Emulsive or milky drinks.* These hold in suspension an oily substance in a finely divided state. Almond milk contains in solution, casein, sugar, and gum; and retains in suspension a fixed oil; agreeing in many of its properties with animal milk.

*Or.* F, *Alcoholic and other intoxicating drinks.* The amount of spirit contained in beer (according to Brande, as given in a table at p. 158), is from 1·28 to 8·88 per cent., and the extract yielded, or its fixed nutritive constituents, are starch, sugar, dextrine, lactic acid, salts, the extractive and aromatic parts of the hop, gluten, and fatty matters; but the quantities are subject to great variation. An imperial pint of good porter gives about an ounce and a half of extract. Dr. Pereira thus proceeds:

"Considered dietetically, beer possesses a threefold property: it quenches thirst; it stimulates, cheers, and, if taken in sufficient quantity, intoxicates; and, lastly, it nourishes or strengthens. Its power of appeasing thirst depends on the aqueous ingredient which it contains, assisted somewhat by its acidulous constituent. Its stimulating, cheering, or intoxicating power is derived either wholly or principally from the alcohol which it contains. Lastly, its nutritive or strengthening quality is derived from the sugar, dextrine, and other substances contained in the extract. Moreover, the bitter principle of hops confers on beer tonic properties.

"From these combined qualities beer proves a refreshing and salubrious drink (always presuming that it is used in moderation), and an agreeable and valuable stimulus and support to those who have to undergo much bodily fatigue. When Dr. Franklin asserted that a penny loaf and a pint of water yielded more nourishment than a pint of beer, it is obvious that he regarded beer merely as a nutriment, and overlooked its stimulating and cheering qualities, of which bread and water are totally devoid." (pp. 415-16.)

Strong ales are richer in alcohol, sugar, and gum, than any other kind of malt liquor.

The term *wine* includes the fermented juice of fruits and saccharine fluids generally, but in a dietetical point of view, liquids obtained by the vinous fermentation of the juice of the grape are most important. The bouquet, or perfume of wine, seems to be an ether (œnanthic), formed by the action of an organic acid on alcohol, during fermentation. All wines are more or less acidulous, from the presence of acetic, carbonic, tannic, tartaric, and probably malic and other acids; but they vary much in this respect. Liebig is quoted, to the effect, that those wines which have most acid have most perfume; and that the acid and characteristic perfumes have some intimate connexion, since they are always found together.

Yet, a few pages onward, we are told that Johannisberger, possessed of a very choice flavour and perfume, is characterized by an almost total want of acidity. Bitartrate of potassa is the most important saline constituent, to which is frequently added tartrate of lime, and tartrate of alumina and potassa. The quantity of alcohol contained in wines varies greatly—from 12 to 20 per cent. in the more ordinary kinds. It varies also in each wine according to its age. The other constituents of wine are chiefly water, sugar, gum, extractive and colouring matters, gluten, sulphate of potassa, and chlorides of potassium and sodium. But the nutritive principles are extremely scanty.

In justification, we may say, of the position in which it is placed, as a dietetical fluid, Dr. Pereira states,—"wine, when used in moderate quantities, as to the extent of two or three glasses daily, proves a very grateful, and to those who have been accustomed to it, an almost indispensable stimulant." It quickens the action of the heart, diffuses warmth, augments muscular force, excites the mental powers, and banishes unpleasant thoughts;—is compatible with perfect health, and many who have thus employed it daily have attained a good old age: its regular use is better adapted for those who lead a life of labour and activity than for the indolent or sedentary. At the same time the two-fold admission is made, that the most perfect health is compatible with *total abstinence*, and that its habitual employment is calculated to prove injurious. "To a person in perfect health, and who has been unaccustomed to it, no possible benefit can accrue from commencing its use." The preternatural excitement which it occasions is followed by a corresponding degree of depression, and the habit of using alcoholic stimulants creates a want for them. After giving Dr. Paris's testimony that "there exists no evidence to prove that a *temperate* use of *good* wine, when taken at *seasonable hours*, has ever proved injurious to *healthy* adults," Dr. Pereira concludes, "*All I would assert is, that, for healthy individuals, wine is an unnecessary article of diet.*" (p. 426.) Wine differs somewhat in its action upon the system from ardent spirits. While those who drink the former are fat, lusty, and plethoric, spirit-drinkers are generally thin and emaciated. The disorders most likely to be induced by wine are those of the digestive organs, of the brain, gout, gravel, and dropsy; while spirits more frequently occasion diseased liver, and delirium tremens.

4. *Condiments* are of five kinds; saline, acidulous, oily, saccharine, and aromatic or pungent. Various organic principles of food escape entirely the action of the primary digestive processes, and are carried unaltered into the mass of the blood. Thus circulated throughout the tissues, they influence the functions of the organs, and probably in some instances, the processes of assimilation. Extractives, tannin, volatile oils, the acrid principles of cruciferous and alliaceous plants, thus escape the action of the digestive organs, retaining their sensible qualities in their progress through the economy: and the impressions they make on the different organs, as also their elimination, may frequently be observed. Thus, they produce effects independent of the nutritive powers of the aliments of which they form a part; these are for the most part stimulant, exciting the action of the heart and arteries, augmenting respiration and animal heat, or irritating special tissues.

The spices, as cloves and nutmeg—labiate plants, as mint and thyme—the fruits and other parts of umbelliferous plants, as parsley or caraway—and some alliaceous plants, owe their powers, as condiments, to volatile oil; but Dr. Pereira is decidedly in error where he refers the properties of ginger to this principle. The acrid principle of ginger is soluble in alcohol, and may be obtained as an extract by evaporating the menstruum. Cinnamon is slightly astringent, and to pimento and cloves have been attributed the property of retarding the fermentation of organic substances, both within and without the stomach. Mustard, cayenne pepper, and common black pepper are among the most powerful local stimulants. The introduction of sugar, oil, salt, and vegetable acid into this part of the work, as condiments, having described them as alimentary principles, and the omission of a substance like cheese, is calculated to create considerable confusion in the mind.

In favour of the use of condiments, it has been urged, that nations almost uniformly employ them, and there is a general dislike by mankind to insipid food, and a desire for sapid qualities; that they serve important purposes in the economy, preventing or arresting ordinary fermentative processes, exciting the secretions necessary to animal digestion—as the saliva, and also the peristaltic action of the intestines; and that their use is founded on an instinct of nature. It must be confessed that the experiments of Beaumont, and the doctrines contained in the work before us, by explaining their modus operandi, and in particular their excitant effects, sanction the views of Cheyne, Cadogan, and others, who regard them as unnecessary, and condemn their indiscriminate use. When confounded together in the form of *sauces*, mostly contrived to please the palate and excite appetite, as "stimulating provocatives," they affect the stomach as alcohol does, and in the healthy state they are non-essential to digestion and afford no nutriment. If it be admitted that in moderation and judiciously selected, they are harmless and even useful, particularly as they occur in food naturally; at the same time it is not to be doubted, that in an isolated state and employed in excess, according to the too frequent customs of the table, they are not only unnecessary but pernicious. These remarks apply to condiments which are not alimentary principles.

The adulterations of food are professedly treated of in Dr. Davidson's work, as indicated in the title-page. These come under three descriptions. 1. Where the nutritive powers are diluted, as in the adulteration of wheat flour with potato-starch, or milk with water. 2. Where one material is substituted for another on account of a difference in price, as the mixture of honey with sugar. 3. Where something positively injurious is combined with food, as lead in the colouring matter of cheese, alum in any notable quantity in bread, or cocculus indicus in beer. Various methods of detecting such adulterations have been employed. By means of the microscope the adulterations of arrowroot with potato-starch may be detected, the latter presenting the appearance as if closely studded with minute globules of mercury. Roasted corn mixed with coffee may be detected by iodine: sloe leaves in tea by their botanical characters. On this part of the subject, Dr. Davidson's work is very incomplete, as looking to its title-page and bulk—how should it be otherwise? With respect to the use of alum in bread, Dr. Pereira remarks:

"Whatever doubts may be entertained as to the ill effects of alum in the healthy stomach, none can exist as to its injurious effects in dyspepsia. Bread which contains alum is objectionable, not merely on account of this salt, but because it is generally made from inferior flour, which, when mixed with yeast and water, and formed into dough, quickly passes through the stage of vinous fermentation, and becomes acid." (p. 311.)

As belonging to this subject also, the soil in which culinary plants are reared, the effects of cultivation, the food with which animals employed at table are fed, and the health both of plants and animals are of the first importance in an hygienic point of view. Dr. Pereira furnishes several illustrations. The ergot of rye, the diseases of wheat from fungi and parasites, and impure milk, may be cited as examples. The qualities of cow's milk are greatly modified by the food of the animal. It is probable that the species of phthisis to which cows are liable, in which it has been ascertained that the milk contains seven times more phosphate of lime than usual, would be attended with injurious results:

"This subject is one of the greatest moment, not only in reference to the frequency of disease in cows, and therefore to the possible morbific character of their milk, but also in reference to the milk of the human subject. I think with these facts before us, it would be highly improper to allow a female with any trace or suspicion of tuberculous disease to suckle. Not that a few grains, more or less, of phosphate of lime in the milk can probably do any injury to the child; but the fact once established, that the milk may be thus altered by disease, leads to the suspicion that some other substances, not yet recognized by their physical or chemical characters, may be in the milk of diseased nurses, and which may have an injurious influence on the child; and the suspicion does not confine itself to those affected with tuberculous diseases; other hereditary or constitutional affections may be attended with altered conditions of the milk. This suspicion is strengthened by the common observation that the milk of different nurses does not equally suit the same child; nor that of the same nurse different children." (Pereira, pp. 255-6.)

Dr. Prout suggests that the diseased liver of the goose obtained by an artificial diet,—the *foies gras*—may produce the fatty degeneration of the liver, in those who employ it; and the organic chemistry of Liebig gives great countenance to such a notion. If fibrin has a vital attraction for fibrin, and albumen for albumen, in a healthy state, we see no reason why the morbid element may not first inoculate the livers of those who consume it, and then convert them into similar masses of disease. Add to these examples, the familiar facts of skin diseases and bowel attacks being produced by crustaceous and molluscous food; that the fat of geese badly fed has sometimes produced symptoms of poisoning; that the same result has followed the use of sausages made of the flesh and viscera of animals, which have acquired deleterious properties by keeping; that the oily matter about the fins of smoked salmon have produced diarrhœa; and that putrid pickled salmon has occasioned death in this country; that Dr. Prout has seen well-marked instances of an oxalate of lime nephritic attack, following the free use of rhubarb, which contains oxalic acid, particularly where hard water was at the same time in use;—facts interspersed in Dr. Pereira's volume—and the importance to the medical practitioner of every circumstance that relates to food becomes abundantly manifest.

The ancients had an exalted idea of the advantages of pure water to the human constitution. They were not only careful in the selection of water, but went to a great expense in preparing it by boiling, both for

4294

drink and for making wines; afterwards cooling it with snow or ice; and public buildings were erected for these purposes. The moderns, it must be confessed, have exhibited a great degree of carelessness on this point. In a foot-note Dr. Pereira states the following fact:

"At the Nottingham Assizes in July, 1836, it was proved at a trial, on which I was a witness, that dysentery, in an aggravated form, was caused in cattle by the use of water contaminated with putrescent vegetable matter, produced by the refuse of a starch manufactory. The fish (perch, gudgeon, pike, roach, and dace,) and frogs in the pond, through which the brook ran, were destroyed. All the animals (cows, calves, and horses,) which drank of this water became seriously ill, and in eight years the plaintiff lost twenty-four cows and nine calves, all of a disease (dysentery) accompanied by nearly the same symptoms. It was also shown that the animals sometimes refused to drink the water; that the mortality was in proportion to the quantity of starch made at different times; and that, subsequently, when the putrescent matter was not allowed to pass into the brook, . . . the fish and frogs began to return, and the mortality ceased among the cattle. The symptoms of illness in cows were as follows: the animals at first got thin, had a rough, staring coat, and gave less milk (from two to three quarts less every day); they then became purged, passed blood with the fæces, and at length died emaciated and exhausted. On a post-mortem examination, the intestinal canal, throughout its whole length, was found inflamed and ulcerated. The water, which I examined, was loaded with putrescent matter, and contained chloride of calcium, (derived from the chloride of lime employed in bleaching the starch.) Traces of free sulphuric acid were occasionally found by one witness." (p. 89.)

Decomposing organic matter in water has long been known to produce dysentery in warm climates; and in this country, in warm weather, the water from our tanks and reservoirs is a prolific source of slighter bowel affections. On the other hand, it cannot be disputed that water, not of the purest kind, is constantly employed by whole families and districts, without our being able to trace to it any injurious effects. Are we then to infer that it does no injury to the constitution? We think certainly not. How many diseases of secretion and nutrition are there, the causes of which have not yet developed themselves? How numerous in all classes of society are the instances of individuals who, without having any determinable disease, acute or chronic, are in a state of health below the hygienic standard? Sir J. Sinclair relates of the inhabitants of Dengy Hundred, Essex, that a very marked improvement took place in their health, in consequence of good water being obtained by sinking a well 500 feet deep. The report of the Poor Law Commissioners for 1842 contains many facts relating to this subject, some of which are quoted by Dr. Pereira at page 95. Fatal dysentery, now rarely met with, formerly prevailed in the navy from the use of impure and putrid water. Mr. Bell, surgeon in Cork, obtained for the troops occupying the barracks, an immunity from the same disease, by substituting spring water for that of the river Lee. It is not only animal matter but mineral substances also that are to be feared—lead for instance, in the waters from our cisterns. Pinel and Schwilgue traced chronic diarrhea and other affections to the well water of St. Salpêtrière, which contained a great quantity of sulphate of lime and other purgative salts, and Parent du Chatelet attributes similar illnesses in St. Lazarus to a similar cause. Dr. Pereira observes—it is by no means improbable that diseases may be induced in the human subject, as in the lower animals, by vegetable and animal parasites derived from water.

"This suspicion is strengthened by the case related by Dr. A. Farre, of a woman who passed by the bowels substances having the ordinary appearance of shreds of false membranes, but consisting entirely of confervoid filaments, probably belonging to the genus oscillatoria. The patient drank the ordinary water which supplies London, and it is not improbable, therefore, she may have in this way imbibed the reproductive sporules." (p. 93.)

If very bad water will produce severe disease and death, the long continued use of that which is less impure may be attended by equally injurious consequences. There can be no reason to doubt, that this is one amongst many causes of physical imperfection in large communities, too much disregarded both by the profession and the public. Excepting alcohol, which might probably be omitted altogether, water is the basis of all our fluid ingesta, and fluid, is of equal importance to health, and as prolific in disease, as solid food. Whether the injurious effects of bad water are counteracted in any degree by the use of alcohol is by no means obvious—even if it be so, we have as it were the resultant of two injurious but opposed forces.

The contents of the entire first part of this volume leave it no longer a question whether man is by nature an herbivorous or an omnivorous animal: he is unquestionably the latter, and capable of being nourished by an animal or a vegetable diet exclusively, because the essential alimentary principles of both are the same, and his digestive organs are suited for the primary assimilation of either. Man is an omnivorous animal, also, because a mixture of animal and vegetable substances appears hitherto to have proved most conducive to the highest grade of physical and intellectual vigour of which he is by nature susceptible. But although this question, so much discussed, may appear to be definitively settled, there is another which has scarcely been considered, except partially, viz., the influence on the constitution, of a more or less highly concentrated state of the various alimentary principles, in our habits of diet. An examination of the facts disclosed of late years by physiology and chemistry will enable us to form some correct opinion upon this subject. As pointed out by Dr. Prout, the best, nay the only type of a perfect diet is milk, the nutritive qualities of which are well described by Dr. Pereira:

"Out of the casein of milk are formed the albumen and the fibrin of the blood, and the proteinaceous and gelatinous tissues. The butter serves for the formation of fat, and contributes with the sugar to support the animal heat by yielding carbon and hydrogen to be burnt in the lungs. The earthy salts are necessary for the development of the osseous system, the iron is required for the blood-corpuscle and the hair, while the alkaline chloride furnishes the hydrochloric acid of the gastric juice." (p. 256.)

Although milk is the only substance prepared by nature for no other purpose but for food, still, many other compound aliments contain several of the alimentary principles, so combined and presented to us by the same bountiful hand, as to leave no doubt of their being intended for the nourishment of our bodies; wheat, and it may be added, the flesh of animals for instance. Although the supply of the latter be small, it is equal in carnivorous animals to prolonged nutrition; as shown by the following fact from Magendie. "Dogs fed solely for 120 days upon a limited quantity of raw meat, preserved their health and weight during the whole period, but when fed with more than three times the quantity of isolated fibrin with some gelatin and albumen, life could not be supported." Wheat, as we have seen, is also a very complex substance; animals may be nourished by means of its gluten alone for an unlimited period, while none of the compounds of protein, animal or vegetable, is sufficient for this purpose. At page 313, we find that notwithstanding bread has been called the staff of life, it does not appear to be capable of supporting prolonged human existence; an assertion made from Boussingault, who came to the conclusion from observing the small quantity of nitrogen that it contains; and from the reports of the inspectors of prisons, on the effects of a diet of bread and water; to which might be added the case of Dr. Stark, who after living on bread and water only, for six weeks, and sixteen days on bread, water, and sugar, found he lost flesh, all the symptoms of bad scurvy with ulcerations occurred, he began to loathe the food, and had he continued this course uninterruptedly he must have died, in the first attempt, of starvation.

On a careful consideration of the compound aliments and their effects, we may very safely affirm that there is something more essential to nutrition than a mere mixture of what we regard as the most important alimentary principles of food. No artificial combination of the principles of milk, previously isolated, would constitute a fluid possessed of all the dietetical properties of that furnished by nature, nor probably of any of those properties in an equal degree; and there must be some reciprocal action of the elements or of their compounds on each other, or some arrangement, which at present escapes our notice. Milk is never so wholesome or nutritious as when sucked from the breast, it is a fluid of great delicacy, the moment it leaves the animal body a halitus is given off, and the atmosphere produces changes in it. So soon as its cream separates, it is no longer the same homogeneous fluid, nor can its constituents ever again be perfectly united. Another important remark suggests itself in considering the dietetical properties of milk,—that if the proportion only of its constituents alter in any notable degree, it becomes unwholesome, and is apt in one way or another to disagree. This is seen in a variety of instances in infants, whose digestive organs become disturbed by an undue proportion of caseum, of butter, and probably of sugar. The milk, also, of one species of animal cannot be so appropriately employed for the nourishment of another species, although every variety of milk is constituted upon the same plan, being subject only to alterations in the proportions of its constituent parts. A consideration of these cirumstances, and of many facts relating to the alimentation of man, leads to the inference, that the most appropriate food for the human constitution is presented to it by nature, requiring only the most simple processes of preparation, in contradistinction to that which is obtained by complicated artificial processes. Although butter, cheese, and whey, are unquestionably wholesome and nutritious aliments, yet an equivalent quantity of the milk from which they are produced, regarding the one and the other, apart from any admixture with which it may be employed as food, is more wholesome and more nutritious, since we cannot employ the former in the proportions adapted by nature, and if we could, the circumstance of their having been separated has deteriorated their dietetical properties.

There is, furthermore, a want of relation in many of the compound aliments between their real nutritive qualities and those which, à priori, they might be expected to possess, from a consideration of the elements and principles which they contain. This is seen in the leguminous seeds, which contain more nitrogen and more of the proteinized principles than the cereal grains; yet for man they are less nutritive. Liebig attempts to explain the fact upon the principle that they are deficient in earthy phosphates, but numerous considerations lead to the inference that this cannot be the only cause. We are inclined to believe that in the case of man, a too-highly concentrated state of the proteinized compounds in his food, apart from the consideration of the absolute quantity which this may contain, is less consistent with perfect nutrition and continued good health, than when these same principles are more completely divided, and diluted with others reputed to be less nutritious; as starch and gum, and in some cases oil. So that, although no doubt the commercial value of wheat, for instance, may be measured by the quantity of gluten entering into its composition, it by no means follows that if employed alone as food, its nutritive value could be measured by the same standard, or that flour, which contains a very large quantity of gluten, is, upon the whole, better adapted for human food than that which contains a fair, moderate average.

If the nutrition of our bodies depends upon certain principles being received into the system, as component parts of our daily diet, how important is it that we should be in possession of correct analyses of every substance employed as food. Questions relating to the diet of individuals or of communities must be referred for solution to these analyses; and in many instances it is upon their accuracy only that any just conclusions can be founded. Bearing in mind that the subject of diet is intimately involved in those of air, exercise, and other hygienic influences, but for the present limiting ourselves to such considerations as belong to the former, the great question to be solved will be—What quantity of the different alimentary principles does any particular system of diet embrace? It is only by the knowledge we have already obtained of these principles, and by reference to these analyses, that we can at all comprehend the results of the habits of the Irish peasantry, of whom it is said there are hundreds who never tasted bread, and millions who live chiefly on potatoes. The potatoes are for the most part accompanied with butter-milk for drink, and frequently with salt herring; hence we have all the staminal principles of Dr. Prout, and all that is essential of the alimentary principles of Dr. Pereira—casein, oil, starch, sugar, salts, and water; the question reducing itself to whether the food as a whole contains a sufficient quantity of these materials. A labouring Irishman will consume from seven pounds to ten pounds per diem of potatoes. If we examine this question in relation to the nitrogenized principles, then ten pounds of potatoes are equivalent to nearly one pound and a half of good wheaten bread, and this, with the protein of the butter-milk, herring, &c., may be regarded as sufficient to supply the wants of the system, and to support "the finest and hardiest race of peasantry in the world." From seven to ten pounds of potatoes contain from fourteen to twenty ounces of carbon, an abundant quantity for the maintenance of animal heat. To the remark, that numerous individuals among the Irish are unable to obtain so large a quantity of food as here indicated, and that this quantity is below the standard of the English labourer, as measured by the quantity of proteinized principles, may be added, that the strength of the Irish is very apt to fail them at a comparatively early age; that the vital force of an indifferently fed Irish labourer is below that of a well fed English labourer; and that experience has shown the former to be particularly obnoxious to diseases of debility. Rice again is said to be "the grand material of food on which a hundred millions of the inhabitants of the earth subsist;" but we well know that these people live not on rice alone; milk forms a common constituent of their food; and independently of this, in proportion as they depend on rice for nourishment, do they consume the greater quantity. In Alexandria, the food of the peasantry, as the Fallaheen women, is chiefly a few lentils, rice, dates, bread made of beans or oatmeal paste, perhaps a little sour Arab cheese, with salt, sometimes black olives, a small quantity of honey, or some gourd or water-melon. These contain all the essential principles of nourishment; but the people get little of them, consequently they are a sickly and emaciated race, and death from absolute starvation is no uncommon thing. Every problem of this kind to be solved must be worked upon chemical data, and our knowledge must turn upon dietetical statistics on a large scale; without which there can be no hope of discovering comprehensive principles. A question again suggests itself here, which does not appear at present to have been much entertained by physiologists: — What quantity of each of the elements or of the staminal principles is necessary for continued health in the various states and circumstances of human existence? This question has been most inquired into in reference to the element carbon; but our information is not yet very precise. Dr. Pereira leans to the opinion (p. 10) that about one pound is required daily, by a hearty adult; subject to considerable variations, according to age, sex, peculiarities of constitution, temperature and density of air, occupation, clothing, &c. Both carbon and hydrogen being regarded as fuel for the production of heat, besides the causes of variation mentioned, the quantity demanded of the former, must depend partly upon the quantity of the latter, available in the economy. This has the greater influence, in so far as that a given quantity of hydrogen, as compared with the same quantity of carbon, combines with double the amount of oxygen, and the heat developed is proportionate to the oxygen employed in these processes of combustion. Again; of the nutritive principles: if it be a fact that some materials in the food, although incapable of affording nourishment of themselves, yet contribute essentially to nutrition when taken in conjunction with others, the question as to quantity can be answered, in the present state of science, only approximately.

### II. OF DIET.

This brings us to the consideration of "the adaptation of aliment to the different wants and conditions of human existence," constituting the second part of Dr. Pereira's volume; comprised in five chapters: I. Of the Digestibility of Food. II. Of the Nutritive qualities of Food. III. Of the times of Eating. IV. Dietaries. V. Of Dietetical regimen suited to disordered states of the Digestive Organs. We shall deviate in some measure in our notice of these subjects from the author's order of arrangement.

I. OF THE DIGESTIBILITY OF FOOD. The notion insisted upon so much by Fordyce, and in fact entertained by his predecessors, that all food must be digested before it can afford any nourishment, has by no means been set aside by any recent discoveries in organic chemistry. All food must undergo certain special changes in the prima viæ before it can constitute chyle, and in all animals it remains there, for a certain length of time, for the purpose. This proposition is true of milk, of the albumen of egg, of the staminal principles of Prout, of the alimentary principles of Pereira —even of oil, which appears to undergo a slighter modification than any other substance, previous to its entrance into the lacteal vessels. The comparative digestibility of the innumerable varieties of food, and of their constituent parts, is of the highest practical importance. As remarked by Cullen, " the powers of the stomach being given, there is a difference in the digestibility of different substances, arising entirely from their differences of (composition and) texture." All writers have made this difference a principal subject of investigation, but their decisions have been too frequently founded upon imaginary qualities and false analogies, and the sciences either of chemistry or physiology do not even now supply many certain or very extensively applicable data. Dr. Pereira considers in this chapter, which is very short, the digestibility of food as " affected by circumstances relating to the foods themselves," and as " affected by circumstances relating to the individual or organism." In so far as these are separable, we shall confine ourselves, under the present head, to an illustration of the former, which we hold to be the more correct application of the term " digestibility."

1. *The cohesive force* is obviously opposed to digestibility, and tenderness of fibre promotes it; hence nature has provided the means of securing minuteness of division by appropriate masticatory organs, and many influences to which food is subject also promotes digestibility by lessening this force. The incipient decomposition of animal food, and violent muscular exertion used by animals immediately before death, act in this way. The admixture of a due proportion of water with substances intended for digestion is most necessary; and nature in this instance again has secured the direct fulfilment of her own objects by providing organs for the secretion of saliva; for, whatever further purposes saliva may serve, this is unquestionably one; and the comparative facility of digestion of various substances frequently turns upon the degree of readiness with which they become incorporated with it. Dr. Beaumont found that tendon, owing probably to its compactness of texture, required five hours and a half for complete digestion in the stomach, whereas, jelly required only one hour.

2. *Chemical constituents.* Some of the alimentary principles are naturally slower and more difficult of digestion than others. The gelatinous principle in many of its forms is by no means easily acted upon in the stomach. It has to be remembered that from ten to fifteen grains of dry gelatin renders four ounces of fluid a tremulous jelly; that substances taken into the stomach require a certain degree of pulpiness to enable them to be acted upon by the stomach, for which purpose their more fluid parts are absorbed, and that semi-fluid substances, as jelly, are for the most part swallowed without insalivation. Hence soups containing much gelatin, the juices of fruits composed of the pectinaceous principle, and even mucilaginous fluids, are apt to remain in the stomach for some time before the absorption of their fluid parts takes place. The oleaginous, as compared with most other alimentary principles, has been understood by all writers to be slow and difficult of digestion. This is shown by the natural antipathy which the tender stomach of the young has to uncombined fat, by oily matter frequently becoming uneasy in the stomach, and by its effect on valetudinarians. "Two members of my family," says Mr. Thackrah, "were annoyed with disorder of the stomach after breakfast. One in particular was uneasy, depressed, and irritable, during most of the forenoon. Plain bread or toast was substituted for buttered toast, and the disorder was removed in the one case and materially relieved in the other." Dr. Pereira insists much upon this point. Oleaginous matters, he states, are first converted into liquid oil in the stomach, then they form a kind of emulsion containing myriads of oily globules, in which form they are absorbed by the lacteals. This process, and perhaps some other unknown change, is affected by the bile, which is found in the stomach during the chymification of fatty substances.

"The popular notion that oily or fatty foods 'cause bile' in the stomach, is not, therefore, so groundless as medical men have generally supposed. From Dr. Beaumont's observations and experiments, it appears that oil is slowly, and with great difficulty, acted on by the gastric juice ; but that the admixture of bile greatly accelerates chymification. Perhaps the alkaline property of the bile partly contributes to this effect." (p. 171.)

Fat is also liable to float on the surface of the stomach as an oily pellicle, becoming odorous, sometimes highly rancid, and exciting heartburn, nausea, and eructation, or at times actual vomiting; and the greater tendency of some varieties than others to produce these effects, depends upon a greater facility in evolving volatile, acrid, and irritating acid and non-acid principles. Mutton fat contains hircic acid, and butter no less than three volatile acids—butyric, capric, and capröic acids; and it is by the changes of composition effected by heat, that oily and fatty substances, after cookery, are more apt to disturb the digestion than fresh and sweet new oil as employed in salads. The observation made by Dr. Combe and others, that the fat of salt pork and bacon is less apt to prove injurious than fresh animal fats, is referred by the author to some change effected in the process of curing. Upon these principles, Dr. Pereira explains the liability of numerous aliments, containing a large proportion of oil, to disturb the stomach. The yelk of eggs, the liver and brain of animals, milk, rich cheese, fried dishes, puddings, cakes, chocolate, the oily seeds, as the almond and walnut, hashes, stews, and many foods in which the oily principle is liable to be taken "in a concealed form," for instance : the indigestibility of the salmon, eel, sprat, and herring, as compared with the whiting and haddock, is owing also to a greater proportion of oil in their composition.

Of the compound aliments, vegetables are held to be slower of digestion than animal foods, and crude vegetables to be more difficult to digest than meat or farinaceous substances; but a discrepancy of opinion exists on the subject. Dr. Cullen gave as a reason, that he had known portions of apple eructated without alteration two days after they had been swallowed, which undoubtedly happens; but Dr. Beaumont found that apples are easily digested in the stomach, requiring only about an hour and a half for the purpose. Imperfect mastication and insalivation accounts for the fact cited, and the structure of the teeth in man and herbivorous animals compared, indicates that vegetable foods require these processes more particularly. It may be here stated, that experiments made on animals by Schultz, led him to the conclusion, that crude vegetables and other indigestible matters are apt to excite a rapid motion of the stomach, by which they are propelled through the pylorus unaltered, in consequence of which they disturb the after stages of the digestive process. The evidence adduced of late, favours the conclusion, that vegetables are for the most part easily digested in the healthy human stomach, and that the argument founded upon experience is in this, as in many other instances, fallacious; the disturbances excited by their use depending rather upon other causes than any inherent quality which prevents or retards their digestion. To this remark, however, there are some exceptions.

3. *Proneness to acidity* is regarded by Dr. Pereira, with all the writers on diet, as a quality which contributes to render various kinds of food indigestible. Hippocrates and Galen, observing that the use of acescent vegetable matters augments the natural acidity of the animal humours, referred many evils to a superabundance of acid; and Sylvius and the chemists attributed half our diseases to an acid acrimony in the system. Very vague notions are even now entertained on this subject. We have, in the first place, to distinguish between the effects of acids taken into the stomach ready formed, as part of the food; and secondly, the effects of acids developed in the stomach from the food. Reasons are given, in the first part of the work, to show that an acidulous alimentary principle is not only digestible and wholesome, but also necessary. Combining with bases in the alimentary canal, the vegetable acids convey saline compounds into the blood, which have their ulterior uses. To acetic acid, in particular, the indigestible qualities of various fruits and other substances have been attributed, yet this acid is a component part of the gastric juice, and one of the best solvents of proteinized substances. Used in moderate quantities in the healthy stomach it promotes digestion. Many substances containing ready formed acids are liable, from other causes, to disturb the digestive organs and produce pain, diarrhœa, and colic. Fruits imperfectly masticated and insalivated, received into the stomach with seeds, flakes of integument, &c., must excite the muscular action of that organ, and their own propulsion into the duodenum, before the necessary changes have been effected. Vegetable matters thus hurried into the small intestines, acting as foreign bodies, are well calculated to create the disturbances just mentioned, which have been too exclusively referred to their acidity. Escaping the chymous changes, they are not prepared for the duodenal changes. The development of gases, increased secretion from the alimentary tunics, and spasms, are the well-known consequences.

But some foods undergo fermentation in the alimentary canal, an important circumstance connected with proneness to acidity. The acetous fermentation has for a long time been recognized, but the lactic acid and viscous fermentations are also known to occur. Dr. Pereira states:

"Lactic acid is one of the substances derived, in part at least, from the food. The alimentary principles which yield it are sugar, dextrine (starch-gum), and gum; those which furnish it with most facility are sugar of milk and dextrine. The acidity of stomach which is produced in some dyspeptics by saccharine substances arises from the development of lactic acid. Milk also is apt to disagree with such individuals, not only in consequence of the difficult digestibility of its fatty constituent (the butter), but also on account of the conversion of its sugar into lactic acid. Both bread and beer contain dextrine, and are the occasional sources of this acid. The tendency which some farinaceous substances, as oatmeal and potato-starch, have to cause acidity of stomach is owing, probably, to the formation first of dextrine, and afterwards of lactic acid." (p. 526-7.)

The production of this acid requires an azotized ferment, which is either contained in the food or derived from the stomach. Tea, coffee and acid juices sweetened with sugar, sweet wines and beer, are thus liable to become acid. Sometimes the process goes on to the viscous or mucilaginous fermentation, and during these changes carbonic acid and other gases are evolved.

4. *The tendency to putrescency* is another quality in food which affects the changes that may occur during digestion. The gastric juice counteracts this tendency, and when putrescent transpositions have actually set in, they are arrested and superseded by the action of this fluid. When the digestive powers are feeble, probably when the gastric juice is scanty in quantity or weak in quality, this tendency in organic matters may take its origin or proceed in the living stomach. It is necessary then to consider the liability of foods to putrescency. Animal substances are more prone to this change than vegetables; pork, as remarked by Dr. Davidson, is very apt to produce nausea and putrid eructations. Some of the ill effects of putrid matter received into the alimentary canal have been alluded to under the head of water, which, when it abounds in decomposing matters, so remarkably interferes with the digestive operations. We may state, in fine, that the usual chemical metamorphoses of organic matter are more liable to occur in certain states of the system, or of the digestive organs, than in others, and that they are superseded by the solutions or series of changes which constitute vigorous digestion in the healthy subject.

5. *Cookery, and the modes by which food is preserved,* here become subjects of medical and scientific interest. Numerous methods have been resorted to by mankind to preserve food; as, drying in the sun, or by artificial heat, smoking, salting, pickling, preserving with sugar, or with ice, or by the exclusion of the air. Although vegetables, as olives, mushrooms, and cabbages, are sometimes salted, animal food is the chief aliment submitted to this process. It appears to be essential that it should be done before putrefaction sets in, but the rationale of the process is by no means, even now, understood. Dr. Davidson states:

"That the animal fibre is hardened and condensed by the process of salting; and that it is impossible by the ordinary methods of maceration, and subsequent boiling, to remove the salt. These facts will readily account for the more difficult digestibility of salted meat. The fibres will thus be less easily masticated and dissolved in the stomach; and the extra quantity of salt being liable to excite thirst, a tendency to overcharge the stomach with liquids will frequently be induced. Salted meat and fish ought therefore to be taken in small quantities by those who have delicate digestive organs. Meat or fish, however, which has been slightly salted, only for a few days, is frequently rendered more tender and digestible by this short process. When kept for many months in salt, it often becomes tainted, is liable to prove unwholesome, or to produce scurvy if used without a sufficient quantity of vegetable food." (p. 91.)

It does not appear that any portion of nutriment is lost; Mr. Donovan having proved by experiment, that the juices usually expelled from meat during the process of salting are mere water tinged with blood; on the contrary, salting frees meat from that which would promote putrefaction. Dr. Pereira leans to the opinion that the preservative power depends, as in the case of some metallic salts, upon a chemical combination formed with the organic tissues. However this may be, hams, tongues, and salted and smoked flesh and fish, are less digestible than fresh food, in consequence of hardness and indissolubleness of texture. The process that hardens and preserves them from putrefaction, before they are taken into the stomach, keeps them from solution afterwards. Pyroligneous acid, containing a small portion of empyreumatic oil, is best adapted for pickling fish and other animal substances. According to Mr. Ramsay, quoted by Dr. Davidson, it is sufficient simply to dip herrings in the acid of 1012 density; and beef may be kept for six or seven months, without taint, by immersion in it for one minute. Another mode of preserving food is that by inclosure in tin cases from which the air has been exhausted, adverted to by Dr. Truman, who states that Sir John Ross and his companions "dined in the Arctic regions on Christmas day, 1831, on a round of beef, some veal and vegetables, all in perfect condition; which had been left with other stores belonging to the 'Fury,' in those high latitudes in 1823;" having been preserved by this method; and that other portions of food brought back to England in 1835, eleven years after it was prepared, remained perfectly good. But we may here remark, that although quite *fresh* as to taste, they have not the flavour or sapidity of really fresh meat. Preserved milk is extremely disagreeable, and in doses of a few ounces might serve as an emetic. Berzelius is quoted, to the effect, that sugar is more frequently employed than formerly for the preservation of meat, owing to a much smaller quantity than of salt being required to prevent putrefaction. Fish when gutted may be well preserved by spreading powdered sugar inside them. Many of the preservative processes, although unquestionably chemical, are not yet satisfactorily explained.

Dr. Pereira thinks the efficacy of sugar, in the preservation of fruits and some other vegetables, is not attributable to its preventing fermentation only, but that "it promotes the solidification of vegetable jelly."

The two-fold object of cookery is to please the palate and to increase the digestibility of food. Taste being intended, with other purposes, to assist in the selection of proper nutriment, when the diet has been habitually simple and the sense continues unalloyed, it is well calculated to insure that object; "for a good taste is the truest mark of good meat and drink, and all meat by how much more savoury it is, by so much the better it nourisheth," saith Roger Bacon. By luxury and abuse this sense is both refined and perverted. The natural desires and aversions belonging to it are to be observed only, for the most part, in infancy and very early childhood. The proper criterion of the qualities of food is afterwards little to be depended upon; we employ the most unwholesome and injurious materials; custom is law, the most acrid and nauseous substances become agreeable, and cookery, from a very simple, is rendered a very complicated art.

Whatever the estimation in which this art is held by the profession, the public, according to Dr. Truman, have appreciated its merits:

"The Romans paid great sums for cooks, who were slaves; and, if they became much celebrated in their vocation, always fetched a high price in the market. In modern times the professors of cookery are liberally rewarded for their services. The principal cook at one of the most celebrated club houses in London, is stated at one time to have had a salary of fifteen hundred pounds per annum—a much larger stipend than is enjoyed by any professor at either of the universities, and far exceeding the value of the greater number of church livings." (pp. 106-7.)

The gratification of a perverted taste, rather than the promotion of health, has ever been the primary inducement for this species of prodigality; and although our modern system of cookery may, upon the whole, excel that of the Romans in simplicity, Apician refinements have still their devotees.

Many of the mechanical and chemical changes effected by cookery are well explained in different parts of Dr. Pereira's volume. A diminution of the cohesive force, and a complete destruction of the organization of the compound aliments seems to be a principal object. The ordinary chemical transformations, by which organic matter passes progressively into the more simple combinations of inorganic nature, are arrested. But the more essential alimentary principles, as protein, oil, and probably gelatin, ought by no means to be changed. The facts of chemistry, as they at present stand, would seem to indicate that any process, having for its result a decomposition of these principles, renders alimentary substances not only indigestible but unwholesome, and destroys their nutritive powers. The processes themselves vary extremely in their effects.

A. *Roasting*, after drying in the sun, is probably the most primitive mode of dressing food, as it requires but little apparatus. Being effected at a very high temperature, a great deal of fat is drained away, and water evaporates; it is necessary to guard against the chemical changes which take place in the fibrin, oil, &c., when meat is overdone; and the juice of underdone meat is almost entirely aqueous. Well-done meat contains less both of water and fat, and is most digestible.

B. *Boiling*. This mode of cookery is considered upon the whole as best calculated to increase the digestibility of food. From vegetables it dissipates the volatile oils, as in the onion, and also gaseous fluids. The grains of fecula in amylaceous substances are broken or split up, the heat and digestive power of the human stomach being insufficient for this purpose. Boiling also coagulates the vegetable albuminous fluids. Gummy and saccharine principles are dissolved, and sometimes injurious principles are extracted; as, for instance, any portion of solanina that may be contained in the potato. In boiling this vegetable, the liquor in the cells and intercellular spaces is coagulated, the starch grains absorb water, swell up, and distend the cells, the albumen forms irregular fibres between the starch grains, and probably also covers them with a thin film, and the cells in which the starch grains are contained separate from each other. In mealy potatoes these changes are complete, in those that are waxy they are only partially effected, hence the latter are less digestible than the former. Analogous changes are effected by cookery in apples and similar vegetables. In boiling animal food, gelatin is dissolved, and part of the nervous fatty matter dissipated; the fatty matters melt, and except when inclosed in close cells, escape; liquid albumen is solidified, as in the egg; and flesh is rendered firmer. Unknown reactions take place during ebullition, and the decoctions, or broths and soups, contain creatine, osmazome, &c., but the essential nutritive principles, fibrin, albumen, some gelatin, fat, and nervous matter remain. Mischief may be done by boiling if injudiciously managed. Albumen if boiled hard is less readily acted upon by the gastric juice and its digestibility is impaired. By prolonged ebullition at 220° Fahrenheit, as in Papin's digester, gelatin evolves ammonia, becomes syrupy, loses its property of forming a jelly, and very speedily undergoes putrefaction. Its nutritive properties are thus deteriorated if not destroyed, and it is rendered less digestible.

C. *Stewing* is in some respects a good method of preparing animal food, the whole of the nutritious matter of the meat being preserved, and its texture softened, but it is required to be done carefully. Soups, hashes, and even stews, if ill prepared, on account of the above-mentioned changes in the gelatin, and of the fat which they contain, are liable to become obnoxious to the digestive organs. It is proved also by Dr. Beaumont, that strong broths and soups, regarded as solutions of gelatin, and incapable of coagulation, have their digestion retarded, until, by the absorption of their watery parts, they acquire a more solid consistence.

D. *Broiling* is analogous to roasting, but effected more rapidly; the outside is liable to be charred, and the fat to be acted upon by the heat.

E. *Baking* differs from roasting, as retaining a larger proportion of the oleaginous principle, also modified by heat; hence baked meat is more liable to disturb the digestive processes.

F. *Frying* is of all methods the most objectionable, the fat in which the substance is placed requires a very high temperature to boil; this, and exposure to the open air, renders it rancid, and promotes the various chemical changes which we have before adverted to; the albumen, as of eggs in batter, is hardened, and the food rendered less easily miscible with the contents of the stomach, for which reasons omelets, pancakes, fritters, &c., are liable to prove indigestible. In fine, any operation in which fat is subjected to a high temperature becomes objectionable, especially where the digestion is weak. This abstract will amply illustrate the scientific relation of cookery to practical medicine, and fully proves, that the more simple processes only are essential. Good cookery in these points of view is conducive to health, and no doubt also to longevity.

These being the principal circumstances, which influence food as respects its digestibility, a remark or two is called for before we enter upon the matter of the second section of the present chapter. That hunger and thirst are not affections of the stomach alone, but belong to the whole system, is true, although many deny and others doubt it. Different states of the system are manifested by these sensations, and by the digestive powers, which in health, for the most part, correspond with the natural appetite. This we apprehend is what Dr. Pereira means by the digestibility of foods being affected by circumstances relating to the organism. We can but regard this as a deviation from the usual logical precision of the writer. Idiosyncrasies may affect the digestion of the most digestible foods, as in the case of the individual alluded to at page 246, in whom mutton acted as a poison. Yet the mutton is not indigestible. However, these states of the system legitimately belong to the inquiry respecting diet, and here we are led to what we believe to be one of the most important results of the discoveries recently made in organic chemistry. Hitherto practitioners in selecting, preparing, and portioning food have for the most part aimed simply at the adaptation of its qualities to the processes of primary assimilation or digestion. The views promulgated by Prout, and more particularly those of Liebig and his school, have rendered more obvious this additional special object in all our dietetical rules,—the adaptation of food to the wants and exigencies of the whole system, or to particular structures, in secondary digestion or assimilation; or for the production of animal heat. Dr. Combe inculcates this adaptation as a principle of practice, but does not enter upon the chemical considerations. By defining more precisely the nature of assimilation, and the materials employed in the nutrition of tissues; and by determining the order and amount of their waste under the various circumstances of life, by means of an examination of the secretions and excretions; we may ascertain what alimentary principles are necessary to be supplied, and are also enabled to form some notion of the quantities required. It is not to be said that many very important results, applicable in the manner we have indicated, have yet been obtained. Still, we are assured that any system of diet which does not include a certain quantity of protein for instance, is inconsistent with that vigour of constitution by which man is enabled to labour in his various vocations; and a diet entirely divested of that principle, if continued for any length of time, is incompatible with the continuance of life. So also, by feeding animals with food having an abundance of the oleaginous principle, or of those materials which are easily converted into oil in the economy, keeping them at the same time in a high temperature and preventing exercise, we can promote the deposition of fat. These instances give countenance to the notion, that by continuing our investigations in this direction, we shall ultimately arrive at practical rules, by which nutrition may in a great degree be regulated, and by which questions respecting diet may be determined, appertaining both to hygiene and therapeutics.

II. OF THE NUTRITIVE QUALITIES OF FOOD. This subject having been fully considered in various parts of the volume, is only summarily treated of in this place. We have seen that aliments differ from each other enormously in the proportions of water to dry matter which they contain, and their nutritive powers must differ in a corresponding degree. The digestible part of the dry matter must be distinguished from that which is indigestible, as from lignin and chlorophyle. The dry material is divisible again into nitrogenized and non-nitrogenized principles:

"For whether the views of Liebig as to the exclusively nutritive quality of nitrogenized foods be or be not correct, it cannot be doubted that the mode of nutrition of substances which are devoid of nitrogen must be different from that of bodies which contain it, and whose ultimate composition is identical with that of the living tissues." (Pereira, p. 453.)

The nutritive value of the nitrogenized part is said to depend upon the relative quantities of the compounds of protein, of gelatin, and other substances containing azote; and the efficiency and utility of the non-nitrogenized part in the production of animal heat, to depend upon the quantity of carbon and hydrogen, and the modes in which these elements are combined. These views, if correct, must originate an entirely new feature in practice. In prescribing dietetical rules, the whole amount of the various alimentary principles, and of the elements of food, that any particular diet may contain, respecting which an opinion has to be offered, must be considered. Looking to the general tendency of these doctrines, the component parts of food must be studied in reference to the wants of the system, and to their nutritive and other effects, not only by the day or week, but for more lengthened periods; and for the purpose of arriving at an opinion as to the suitableness of any given diet, we must determine the amount of carbon, hydrogen, nitrogen, protein, &c., which it contains. For this purpose accurate and well-constructed tables of the component parts of different kinds of food will prove extremely useful. Dr. Pereira's work contains several such, that at page 454 showing the average quantity of dry matter, moisture, carbon, and nitrogen, in various alimentary substances of commerce, may be particularly referred to.

III. Rules of Diet. A matter of some consequence is this: heedless persons, and those who are unwilling to have the gratification of their appetites controlled, or restricted within reasonable limits, frequently urge that dietetical rules are useless and unnecessary. They maintain this assertion by an appeal to their own experience, bringing in support of it the cases of individuals who have lived to a very old age, of whom Parr and Jenkins are cited as examples, although erroneously, as having done so without following any particular rules of health. Such arguments are frequently employed against the advice and prescription of the medical practitioner. In support of the same assertion also, a doctrine taught by Celsus is not unfrequently mistaken even by medical men; "modo plus justo, modo non amplius assumere." But this is itself a rule, and as Hallé and others have interpreted it, by no means a sanction to excess or carelessness of any kind. Without lending any countenance to that kind of over-solicitous attention to the health which belongs to a luxurious age, as one of its refinements, it may be fairly questioned, whether any man above a certain class of society stands the slightest chance of attaining a good old age, who does not adopt rules of diet adapted to his age, particular constitution, occupation, and habits of life. As remarked by Sir J. Sinclair, savages have their rules, which are instanced in the self-denial of the American Indians, and of the ancient Germans, with a view to preserve their strength. "Peasants, labourers, and other hardworking people are placed in that situation where few rules are necessary, because their whole lives are a series of indispensable attentions to air, to exercise, to moderation as regards diet, drink, &c. . . . Rules they do observe, and for the most part every old person will be found to have his maxim of health." Plutarch, Galen, Cornaro, the Cardinal de Salis, all of whom attained a very advanced age, had their precepts of health, which are still extant. Not only for the purpose of relief or cure when the functions of life have become deranged, and the organs diseased, but even in the absence of these, for the preservation of health, no reflecting person can doubt the efficacy of rules. The mistake has been in endeavouring to lay down one principle for all mankind, and in prescribing general rules without a sufficient regard to individual constitutions.

IV. Quantity of Food. In the present state of our knowledge it is impossible to offer any fixed standard by which to determine the quantity of food required for any given individual. At the same time, the fact that medical and other official authorities are daily called upon to form diet-tables for large classes of their fellow-men in hospitals, prisons, workhouses, schools, and other public institutions, the importance of arriving at the closest, possible approximation to such a standard becomes self-evident. But in pursuing this object we must be careful, as Dr. Davidson remarks, neither to lose sight of the many circumstances which modify the quantity of food required, nor to confound the quantity barely sufficient to maintain existence with that required to ensure vigorous health and longevity. Of the circumstances by which the quantity is affected, Dr. Davidson mentions two only—absolute bulk and exercise; in touching upon which, he alludes to the exceptional cases in which very slender men far excel their bulkier brethren in their daily alimentation. If Liebig's principles are to be depended upon, the amount of food required for any individual ought to bear a relation—1st, to his absolute bulk; 2dly, to his expenditure of vital force, as measured by the amount and kind of bodily and mental exertion which he undergoes, and the length of sleep; 3dly, to the expenditure of caloric as influenced by climate, season, clothing, artificial heat, and evaporation at the surface of the body; 4thly, to the growth and decay of the body in infancy and decrepitude.

A careful consideration of the doctrines propounded by Professor Liebig in his late work, and of a vast number of facts relating to the variable habits of mankind as respects diet, and of the effects of these habits, leads to the inevitable conclusion that, especially at the period of what may be called stationary adult age, as in different species of animals, so in different individuals and varieties of the human species, there exist fundamental differences in the constitution, as to the degree of rapidity with which the molecular changes take place in the vital tissues. These differences may be hereditary, constituting an important feature in the various temperaments, or they may be acquired during the periods of growth and early nutrition; but, whatever their origin, the more or less rapid metamorphoses which they imply characterizes in part the individual constitution. Without going so far as to agree with our friend Mr. Grisenthwaite, that there is no such thing as waste and reparation in the animal body, we think that in some constitutions the quantity of matter changed daily is very small indeed. In others it is much more considerable, and the differences between different individuals in this respect is much greater than has been imagined. One man, accordingly, requires a much larger quantity of the staminal or alimentary principles of diet than another of the same weight. This is true of the living structures as a whole, and even particular structures or tissues differ from each other in the same respect, laying the foundation probably for particular idiosyncrasies and habits.

The proportionate size of the different organs has also its influence over the quantity of food which an individual consumes. Whether from original conformation or from habits of abstinence or repletion, the human stomach varies greatly in this respect. Our own observation, in repeated post-mortem examinations, has convinced us that an enormously large stomach is by no means conducive to longevity. One of the largest we have ever seen was in a woman who had been an enormous feeder, was extremely fat, and died at the age of fifty-six, suddenly,—being anæmic, her muscles blanched, and having a large cholesteric calculus in the gall-bladder. We have observed the stomach of a very moderate capacity in old people. The absolute capacity of the stomach must have something to do with the quantity of food required, and although the size of this organ cannot be accurately determined during life, its property of distensibility and the habits by which individuals act upon this property must be regarded in rules of diet. So also Dr. Davidson must be correct when he says:

"If one man have larger lungs, greater extent of skin, and more muscle than another man, he of the greater capacity must consume more carbon and nitrogen in the support of these functions; and hence will require more food containing these elements. Greatly, however, as some men differ from others in natural size, this is as frequently owing to accumulation of fat; and a man may thus nearly double his weight without increasing the capacity of his lungs, the size of his muscles, or the number of his cutaneous pores. Men who have thus increased their weight by corpulency do not, for this reason, require the same amount of food as those of similar size who are favoured with no such covering; and it is possible that the fat when once formed is allowed to remain partially dormant; and hence its actual weight is supported by less food than was required for its formation." (p. 27.)

It may here be remarked, that the cases of Cornaro and of the eastern Christians who retired from the persecutions to the deserts of Egypt and Arabia, living to a great age upon a few ounces of bread and herbs with the pure element for drink, are unquestionably instructive, and should not be lost sight of. But erroneous inferences have too frequently been drawn from them, and the sources of error are in the omission to allow for the effects of climate, age, exercise, or rest, and certain habits. Probably many of these individuals lived to be 115 or 120 years of age. The facts illustrate the principles developed by modern science: they took very little exercise, were at such an advanced age that the change of matter was extremely slow, and living in warm climates as saints and hermits are wont to live, in the shade and quietude of their caves and grottos, they required very little fuel for animal combustion. The diet suited for this combination of circumstances has been ill-chosen as the prototype of that which is necessary for mankind in general. The principles of science appear to be equally well borne out by the numerous instances of longevity in various parts of England, in which the diet has always been plain, simple, and abstemious, but as required by the greater coldness of the climate and the greater activity of its inhabitants, much more abundant than in the former cases.

These various circumstances serve to explain how it is that many individuals not only suffer no injury from a large quantity of food, but absolutely require it. How others, on the contrary, preserve health on the sparest diet. They show that one constitution demands more or less azotized or non-azotized or oleaginous food than another. By taking into consideration these modifying circumstances, many seeming discrepancies, respecting the diet employed by different people, will vanish. These circumstances determining the natural appetite ought to be taken into the account when vicious customs, or the causes of disease, or anything whatever, has occasioned the sensations no longer to be depended upon, and has given origin to the necessity for laying down some rule as to the quantity of food to be taken by an individual; and in the construction of dietaries.

The absolute and proportionate quantity of liquid necessary for the human constitution is another important consideration. Hoffman laid it down as a principle that three parts of the ingesta ought to be fluid in proportion to one of solid; founding this upon the experiments of Boyle, who had ascertained that the blood contains three of fluid to one of solid constituent parts. Liebig makes the proportion of dry matter in the blood twenty per cent. Different writers on dietetics have taken very different views of this matter. To twenty-four ounces of solid food Cheyne allowed forty-eight ounces of fluid. Arbuthnot attached great importance to the principle of keeping the blood and other fluids in just proportion to the capacity and strength of the vessels, there being more danger from too small than from too great a quantity; but too much fluid weakens the activity of the digestive powers. Several authors have urged, we believe with propriety, the inconveniences of too little fluid, in producing costiveness, and leading to a faulty composition of the blood in the direction of a too great proportion of the red corpuscular part to the serum. The rule laid down by Hoffman is probably not far from right, but the proportion should be calculated upon the dry materials of food, not as it is commonly used. Thus dry rice when prepared for table combines with so much water that drink is hardly required with it; but bread, containing no more than forty per cent. of water, would require above its own weight, to be furnished either by the salivary glands or in the form of drink, to render it sufficiently fluid for the different stages of digestion, and for its conversion into chyle and blood. Any rule of this kind can be admitted only in a very general way, for when, from any cause, a greater quantity of fluid than of solid material is dissipated, as happens under a variety of circumstances, more fluid must be taken into the system. There are two classes of cases in which thirst is induced; those just mentioned; and where materials foreign to the blood, as poisons, or an excess of some of the healthy constituents of the blood, as of its salts, are received into that fluid. If the proportion of solid to fluid be duly preserved in the ingesta, and the food composed of wholesome alimentary principles only, in a fair proportion, the balance between the ingesta and the ejesta being moderately well preserved, the sensation of thirst and the necessity for large potations would seldom, if ever, recur. A close examination of the habits of diet among individuals in our own circle of observation, has led us to the conclusion, that the most robust and active consume from twenty to twenty-four ounces of dry material, and from sixty to eighty ounces of fluid daily. The quantity and proportion are subject to great variations, both within and without these limits; but the most frequent error committed, particularly among females, would appear to be an excess of liquid as compared with solid food. It is also unquestionably true, that an inordinate quantity of fluid received into the system is less injurious, in consequence of the almost incredible rapidity with which, under favorable circumstances, the excess transpires.

v. AGE. For reasons before alluded to, the proportionate quantity of food required by the system at different ages differs considerably. During the periods of infancy and growth, the increment of the tissues, the additional loss of caloric, owing to increased surface in proportion to the bulk of the body, and the very great comparative muscular activity in youth, are so many causes in operation to demand a greater proportionate quantity of nourishment. Physiological considerations lead us to infer that a greater proportion of water to dry matter, and of non-nitrogenized to nitrogenized aliment, is demanded in the earliest periods of life. The nourishment requires also to be well adapted to the digestive powers, hence the earliest food is furnished ready prepared by the nurse. A principle laid down by Lord Bacon seems to apply to this part of the subject, that it is a general law of organized nature, for those species and individuals that are slow in attaining maturity to live the longest, nature finishing her periods in larger circles. If this be admitted, an over nutritious diet during the period of growth may have the injurious effect of bringing the constitution forward too rapidly. At the same time, every practical man will agree with Dr. Pereira that,

"Of the ill consequences of defective nutriment we have, unfortunately, too many instances continually presented to our notice. Irritable bowels or diarrhea, tumid abdomen, mesenteric disease, wasting, and fever, are the ordinary and obvious effects. They frequently follow the continued use of pea-soup and potato-stews, dishes which are in common use at poor-houses and other establishments for pauper children. Scrofulous and strumous diseases, marasmus, rickets, distortions, and pot bellies, so commonly met with among children of the poor, are referrible, in part at least, to food defective either in quantity or quality, or perhaps in both. I think it will be found that more than two thirds of pauper children are strumous. They derive this condition in part, perhaps, from hereditary tendency; but partly also, as I believe, from defective nutriment. To the same cause also is ascribable their inferior development. If the children in poor-houses be examined, they will be found for the most part, smaller and shorter for their age, more frequently distorted, and more readily fatigued, than the children of the middling and higher classes." (pp. 473-4.)

In adult life, in so far as the habits and locality of individuals are stationary, there is greater propriety in establishing fixed rules of diet than in the periods of development. A balance between decay and reparation exists, and probably during many successive years these processes are pretty uniform in degree. With change of locality, of season, and of habits, variations both in the quantity and quality of sustenance are required, but in the ordinary routine of life these may be kept within very moderate limits. As age advances the change of matter takes place more slowly; the powers of the body and the capability of labour and exertion, and in a proportionate degree the appetite for food decreases. We have estimated the quantity of aliment, and the proportion of dry to liquid material, consumed habitually by several old persons in good health. It varies from forty to sixty-five ounces, and the solids, but more particularly the nitrogenized alimentary principles, are more than proportionably diminished. An old lady of ninety, now living, walks about a mile daily, and enjoys uninterrupted good health with the possession of all her faculties; her daily diet is, upon an average, five ounces of solids and thirty-two ounces of drink, including eight ounces of porter and two glasses of wine. The cases of longevity published confirm these observations, and under every practitioner's eye, individuals of both sexes, who have arrived at a considerably advanced age, glide along the stream of life in the enjoyment of health, upon a very moderate supply of nutriment. So long as the supply and the expenditure as nearly as possible balance each other existence continues. It is a common expression applied to a bed-ridden octogenarian "he lives upon as little as would feed a sparrow." Let the balance be disturbed and existence is cut off. An increased quantity of nutriment destroyed old Parr, and two ounces in addition to his daily solid food nearly killed Cornaro. Many old men are prematurely cut off by exhaustion, "their spirits lead them beyond their strength." If neither of these causes operate, the appetite for food gradually diminishes and the flame of life wavers and flickers and is finally extinguished, like the spark of a candle. These phenomena are beautifully developing themselves by the light which science is now casting upon the economy of animal life.

VI. CLIMATE AND SEASON. Great differences in the habits of mankind, as respects food, clothing, and mental and physical power and activity in the different countries and regions of the earth, and indeed the habits of animals, have been referred to, for the purpose of testing Liebig's doctrine of the source of vital power and that of animal heat, as contradistinguished,—the one being from nitrogenized compounds and the change of matter in the vital tissues, the other from the combustion of carbon and hydrogen. We have not seen the camel referred to in illustration of this subject. A large camel will carry from seven to twelve hundred weight on its back at the rate of thirty miles a day, subsisting for eight or ten days together upon dry and thorny plants and other scanty herbage. Here is a great expenditure of force, and we may presume little assimilation of nitrogenized food. If this were the whole truth it would militate against Liebig's views; but we learn that from about the period mentioned, more nutritious aliment is required, and that various artificial and, no doubt, nitrogenized preparations are given to the animal; the herbage referred to may contain a considerable portion of proteinized aliment; and it remains a question, to what extent the vital tissues become wasted under this expenditure of power. The provision for water is complete, the stomach being constructed with a cellular reservoir capable of containing a quantity sufficient for many days' consumption; and the boss on the back, which consists entirely of fat, supplies carbon and hydrogen; becoming wholly absorbed if the supply of food be not maintained under the fatigue described. Turning our attention to man:—The aboriginal inhabitants of the isles and warmer parts of America were remarkable for the smallness of their appetite for food; the earliest historians of the country describe their constitutional temperance as exceeding the abstinence of the most mortified hermits:—one Spaniard is said to have consumed more food in a day than ten Americans—their habits were those of indolence and total want of occupation. Hence,—debility of frame, a vacant inexpressive countenance, listless inattention, the mind being almost totally inactive, the emotions and efforts few and languid—the passion even of love almost inert. In the inhabitants of the more temperate regions of Chili and North America, and those districts where the people of America lived by hunting and were obliged to exert unusual activity, the appetite has sometimes struck observers as being voracious. Let us glance at the case of the Hindoo, which has been cited, although by no very philosophical writer, against the doctrine of the origin of animal heat and vital power. The Bengalee lives in a tropical climate upon a rice diet, and this, it is said, is essentially a carbonaceous diet containing very little nitrogen, yet for many months of the year the production of very little heat is required in his economy, and he goes through great muscular exercise. It can only be minds little imbued with the facts of science, which would employ these imperfect observations as arguments conclusive against the doctrines before us. As of the food of camel, it may be asked, what is the amount of the proteinaceous principle really contained in that of the

Hindoo? If the Bengalee actually consume a large quantity of fuel for organic combustion, does he not expend a great deal of caloric? Every possible means is resorted to by him for the purpose of reducing the temperature of the surface of the body and that of the surrounding atmosphere. If his food be highly carbonaceous it is also extremely aqueous, for he drinks much; he also wears little or no clothing, exposes himself much to the open air, and evaporation goes on at a great rate. Moreover, it is not to be doubted, that the food employed in hot climates frequently contains an excess of carbon and hydrogen, so far as the wants of the system are concerned. This is shown by a disposition to obesity. From Mr. Laird's narrative of an expedition up the Niger, we learn that all the party had a disposition to get fat, not eating half what they were accustomed to in England. So also Hindoos are apt to get fat on rice, and Negroes on the excessive use of sugar. This excess of carbon and hydrogen in the food also manifests itself in the diseases of hot climates. The Boothian, with other inhabitants of the polar regions, on the contrary, not only eats enormously and is accustomed from childhood to a large quantity of whale oil or blubber for food, but digs his grotto in the snow, and wears a double or triple garment of seal or reindeer skin,—he husbands most carefully his animal heat. We recommend those who may be sceptical on this point to apply to Mr. Richard King, of the Ecthnological Society, for an opportunity of wearing an Esquimaux dress for half an hour. We in England could not endure it in the coldest weather.

These facts apply to the different seasons and changes of external temperature. It is true that in our variable climate we do not find a whole population ravenous on one day, and totally incapable of consuming food on another, to correspond with every thermometrical and barometrical change of the atmosphere. The difference in the quantity of caloric extricated under these changes is frequently much less than would, à priori, be imagined. Warm clothing, warm beds, good fires, and heated apartments may equalize the summer and winter demand for the elements of respiration in our food. Diurnal changes may be too transitory to interfere with our habits of returning appetite and repletion. Yet common experience proves that much exposure to cold produces a necessity for increased muscular activity, and with this, a greater demand for food; and a sultry summer day diminishes the appetite and renders the digestion languid. Insufficient diet with exposure to cold, and excessive eating and drinking of hydro-carbonaceous aliments in hot weather, are equally liable to induce pathological conditions of the system, and fatal diseases.

VII. Times of Eating. Some authors have advocated no fixed periods for meals, and have been in favour of leaving every one to eat and drink as his convenience or his sensations might dictate; but the principle of periodicity in the human constitution, if properly considered, is conclusive against the propriety of this practice. In infancy and youth, as regards time as well as quantity, it may be granted that regularity is less required, but in adult age and advancing years, the necessity of stated periods for meals is unquestionable. Darwin well remarked on this point, that the periods of hunger become catenated with certain portions of time or degrees of exhaustion, or other diurnal habits of life; and if hunger be not relieved by taking food at the usual time, it is liable to cease till the next period, or other habits recur. There has been but little agreement as to the number of meals necessary. That the body may be regularly supplied with nutriment and fuel, the intervals should not be too great, experience having amply proved that injurious consequences result from inattention to this observance. That the food may be properly digested, the intervals should not be too limited. Dr. Beaumont's table, reproduced by Dr. Pereira, shows that different aliments require from one to five or six hours, and a moderate meal about three hours and a half for complete digestion in the stomach. This organ should remain empty for a time before it is again filled, as indicated by the fact that the appetite does not immediately return. The explanation which suggests itself to us of this circumstance is, that it is necessary the after stages of primary digestion in the duodenum should not be interfered with by fresh accessions of chyme before they are complete. These considerations enable us to arrive at some conclusions as to the proper number of meals. During the luxurious periods of the Roman empire, five and sometimes six were taken daily, and the more wealthy classes of old England had four and sometimes five meals a day. Long experience has demonstrated that this frequency of eating is inconsistent with the highest point of intellectual and physical vigour of which the human constitution is susceptible: not to say, with the continuance of health. It prevents the recurrence of natural appetite, and is usually associated with a pampered taste, to be satisfied only by a complication of food, condiments, physic, and poisons. The most robust among the peasantry and labouring population continue so on three good meals a day, taken at regular intervals. It is also a fact that some of the most healthy and vigorous people, particularly in the middling and upper classes of society, habituate themselves for years to but two substantial meals daily. Observation convinces us that among the latter, the sum of vital force expended in bodily labour is less, although that expended in mental exertion through the involuntary organs, as in the cares and anxieties of life, may be greater than in the former class. In the letter from a physician, recorded by Sir J. Sinclair, it appears that the very old men in the highlands of Scotland, of whom M'Alpine reached 119 years, took but two meals a day; a great many of the poorer Irish divide their daily food into two portions. We think upon the whole, that Drs. Pereira and Davidson are judicious in determining that three meals in the twenty-four hours is the best general rule to be laid down for the population of this kingdom; admitting, however, of numerous exceptions, as for infancy and old age; and as respects the former, not only are a greater number of meals required, but from the moment of birth, the natural tendency to periodicity should be respected in the customs adopted by the nurse, in feeding both herself and her infant.

VIII. HABITS IN REFERENCE TO MEALS. The propriety of taking exercise or rest of body and mind immediately before, during, and after meals, is an important consideration. The system ought not to be in a state of excessive fatigue or exhaustion at the period of taking a heavy meal, and deep thought during meals is most inimical to the digestive process. Dr. Pereira shows that there has been much difference of opinion upon the subject of sleeping after taking food; he leans in favour of doing so, after a principal full meal, as dinner. Active exercise, it is stated, is prejudicial to digestion, and various evils have been traced to it, as diseases of the heart. The habit of animals has been used as an argument; but we may ask, because the dog or the serpent take immense quantities of food at long intervals, gorging themselves to repletion, and then lie down and sleep, is man also to do so? According to Seguin's experiments, when exercise is taken after a meal, more than double the quantity of carbonic acid gas exhales from the lungs, showing the increased combustion of fuel and production of heat under the combined influence of food and exercise. Dr. Beaumont ascertained that moderate exercise heightens the temperature of the stomach and promotes digestion in a very marked degree. Are we prepared to say this is injurious? The evils of violent exercise, or of intense thought, with an over-distended stomach, are not to be doubted. But we are inclined to respect Aph. 36, sect. 3 of Sanctorius, "That is the most healthful amount of food, when after eating the body performs whatever it has to do with the same agility as if it were fasting." So also Cardan: "The true measure of eating and drinking is, that a man shall feel no fulness or weight in his stomach, but shall be able to walk or write immediately after meals:" and Lessius, "He who eats or drinks such a quantity as renders him unfit for any exertion of the mind to which his profession calls him, has certainly exceeded, and ought to retreat. And he who in bodily labour or exercise was active and nimble before meals, if he becomes heavy and dull after meals, has certainly transgressed; for the true end of eating and drinking is to refresh, and not to oppress the body." Aphorisms, which we must confess, notwithstanding they are contrary to the practice of the majority of individuals of the upper classes, who will plead personal experience to contradict them, being confirmed very strongly by the experiments to which we have referred, we believe to be framed on a rational foundation.

IX. Dietaries. The introduction of this chapter, which contains the various hospital, prison, and workhouse scales of diet, constitutes a peculiar, and it must be admitted a very important feature, in Dr. Pereira's work. There can be no doubt, that if preceding writers had paid more attention to this part of the subject, much misery, disease, and loss of life would have been prevented. A consideration of the effects of dietaries on large numbers of individuals is calculated to bring our principles to the test, but, on examination of the chapter before us, we are bound to say that the data it contains, by which conclusions might be arrived at upon some of the more important points, are extremely few and defective. This is in part attributable to a circumstance before remarked on, the want of a philosophical foundation, upon which questions respecting diet might be determined. The time can scarcely be said to have passed, when one learned physician who had allowed his patient a little stewed beef, felt aggrieved at his brother in consultation unwittingly ordering a mutton chop. An unlucky apothecary allowed his patient an oyster or two.—"Oysters!—Mrs. General G——, I tell you oysters are your destruction," exclaimed the physician on the following day. One of the old school, now dead, had ordered a patient bread and water for breakfast; after enduring this for some time, the lady complained that she could not go on, and must have something stronger. "Then, madam, you may add a little toast to your water!" was the answer. The truth is, our prescriptions have been too frequently based upon some real or imaginary trifling difference in the digestibility of foods, or given at hazard, without any reference to the amount and kind of nourishment required, or to the proportions of the different alimentary principles, or the special objects to be served in the economy. Upon equally vague and defective principles have dietaries been framed for public institutions, and most deplorable, as is now well understood, have been the consequences.

In the dietaries for children we observe that the quantity of substantial food for growing boys, for instance, sanctioned by "le ministre de l'Intérieur" in the public institutions of Paris, greatly exceeds and in some instances doubles that allowed in our own establishments. In the Foundling Hospital in London, the substantial diet for a boy of eight years old, is upon an average a little under 19 ounces daily, to which is added nearly 14 ounces of milk. In the Foundling and other hospitals of Paris, for a boy of the same age, the substantial food is about 30 ounces, to which is added about 30 ounces of broth, soup, and wine. The diet for the younger children in the Foundling is not so good, but after nine years of age it is more nearly equal to that of other metropolitan institutions. In connexion with this subject we have been furnished with an important fact by Dr. G. O. Rees, assistant-physician to Guy's Hospital. The average weight of a given number of boys in the above hospital, whose ages are under nine years, is six pounds each, less than that of the same number of boys of the same age in the Caledonian Asylum; the amount of nutritious food allowed in the latter establishment being much greater than in the former. There are no doubt other causes in operation, but the fact deserves the closest attention.

In the English navy each man is allowed daily from about forty to forty-five ounces of solid food, greatly varied, with a gallon of beer and some tea and vinegar. Dr. Pereira considers this "most ample, though not excessive." There are some anomalies in the different scales:—eight ounces of fresh vegetables, for instance, are made equivalent to twelve ounces of flour. The former contain, probably, some saline matter or other principles essential to nutrition, which are absent in the latter; but, measuring the nutritive powers by the whole amount of dry matter, eighty-six, instead of eight ounces, would be equivalent to the given amount of flour; and measuring them by the quantity of nitrogen afforded, 105½ oz. would be the equivalent. Notwithstanding this, the quantity of food being abundant and the quality good, a high degree of health is enjoyed in the royal navy.

On the dietaries for paupers we meet with the following announcement: "It has been very properly stated by the Poor Law Commissioners, that in the dieting the inmates of workhouses, the object is to give them an adequate supply of wholesome food, not superior in quantity or quality to that which the labouring classes in the respective neighbourhoods provide for themselves." Six dietaries have been adopted by the commissioners for use in poor-houses. The average amount of solid food daily, for able-bodied paupers, is about 25½ ounces for males, and nearly 22 ounces for females. Bread, cooked meat, and potatoes, form the most important items; beer is not permitted unless specially ordered by the surgeon; and at sixty years of age and upwards, 1 ounce of tea, 5 ounces of butter, and 7 ounces of sugar per week, in lieu of gruel for breakfast, may be allowed if deemed expedient. In justification of this scale of diet, we are informed that an agricultural labouring man consumes pro-

LIBRARY OF THE

Orleans Parish Medical Society bably no more than 20 ounces of solid food daily—bread and meat—but including not quite an ounce of the latter. And, also, that at a meeting of the chairmen and vice-chairmen of the twelve East Kent Unions, it was unanimously declared the diet answered well, no alteration being desirable.

Dr. Pereira remarks of the prison dietaries, "It will be perceived that the conclusions which the inspectors have arrived at accord with the principles which I have advocated in this work." The diet is regulated according to the length of imprisonment, sex, discipline, and the additional punishment of hard labour. The solid food for prisoners at hard labour, for terms exceeding three months, is, for males, a little more than 36 ounces, and for females a little more than 26 ounces.

To a certain extent only, as it appears to us, have the inspectors of prisons acted upon the principles embodied in the work we have been reviewing; founded on the chemistry of organic life as at present understood on the continent, and in this country, by a large and increasing class of the medical faculty. In this point of view, the "Reasons of Dissent as to the Scales of Diet," assigned by Mr. F. Hill, and very candidly inserted by Dr. Pereira, are deserving of attention. Mr. Hill urges that there is great want of information as to the quantity and kind of nutriment required for health under the various circumstances of life, and also as to the quantity and kind of nutriment contained in aliments. He objects also to the principle of giving prisoners less food for the sole reason that the imprisonment is to be of shorter duration. Viewing this subject as a whole, we may add, that we are *very* sceptical as to the sufficiency of the pauper dietaries. In the first place, the whole of these dietaries are not so much varied as with advantage they might be. At the Pentonville prison, Dr. Rees employs cocoa and treacle, and we know no valid reason why these and many other cheap commodities should not be introduced into all prisons, but more particularly into poor-houses. The quantity of food for able-bodied paupers is upon the whole less substantial than that allowed by Cheyne, after the example of Cornaro, which has been regarded by all the most judicious writers as too spare a diet for this climate. Dr. Starke starved on 26 ounces of bread per diem. As to the quantity of food obtained by agricultural and other labourers, we have to remark, that in the county of Wilts, according to Mr. Chadwick's report, the average age attained by the labourer and his family is thirty-three years, by the farmer forty-three years, and by the gentry, fifty years. In Whitechapel the average age attained by the class of mechanics and their families is twenty-two years: tradesmen twenty-seven years: the gentry forty-five years. A general law of this kind appears to obtain. Several causes for this are in combined operation, but we apprehend no doubt can exist, that food, deficient in quantity, or injudicious, or bad in quality, is one of the principal. "The labouring classes," says the Report before us, "become old the soonest, and the effects of the unfavorable influences in the adolescent and adult stages is shown in the smaller proportion who attain extreme old age; and also in the periods of the deaths of heads of families of this class." (p. 161.) In the union poor houses several of the most efficient causes of premature decay and death, as close habitations and uncleanliness, exposure to vicissitudes of temperature, malaria, inordinate labour, the use of intoxicating liquors, &c. &c., are done away with; so that, except so far as diet is concerned, this law ought to cease, or to reduce itself to a very narrow scope. Accurate statistical records and time will prove to what extent this takes place. The same test will exhibit the frequency and effects of diseases of debility. In the mean time, it behoves those in authority to consider well their scales of diet, since these must become more than ever amenable for the contingencies of the union poor-house.

The best of the dietaries for able-bodied paupers of the male sex affords about 8 ounces of carbon daily, and for the females 6½ ounces. We do not know how Dr. Pereira reconciles his approbation of these dietaries with his statements in the first part of the work, as to the quantity of carbon consumed by individuals under different circumstances. There is no great amount of hydrogen in excess in the different scales; accordingly, if anything like a pound of carbon, or its equivalent of carbon and hydrogen, is necessary, in this cold and variable climate, for the maintenance of the proper temperature of the human body, these dietaries must be most defective. The carbon is considerably less than that allowed for the prisoners in the house of arrest at Giessen, who are deprived of all exercise. (Pereira, p. 12.) In reference to this point, it is not absolute starvation that we have to look to as the most obvious and immediate result of the system of diet, but the occurrence of diseases which supervene a low power of resisting the effects of atmospheric vicissitudes. Artificial warmth might in part obviate this tendency, but the temperature of the union workhouse, and the day and night clothing employed, would not place its tenant upon a par in this respect with the individual living at ease in a tropical climate; and if it did, the continual exposure of the respiratory organs to the action of the external air, must, as we should imagine, be attended with the results to which we have referred.

CHARITY HOSPITAL, MEDICAL LIBRARY

The fact of inherent differences of constitution in different individuals, is calculated to show the utter absurdity and cruelty of restricting a body of men, whether paupers in a workhouse, or the inmates of a prison, to a uniform minimum allowance of food. This has been found out experimentally, to the bitter cost of large classes of our fellow-creatures. At the same time it will no doubt be held to be impracticable in our institutions, to apportion to each individual the quantity of food which the special circumstances of his constitution may require. How, it may be asked, are we to judge of the weight and bulk of each individual, of his habits of bodily and mental activity, sleep and watchfulness, of his respiratory organs, and powers of producing heat, of all the circumstances of external temperature and artificial warmth, of his previous habits, of the relative size of his organs, and of the degree of molecular activity in his vital tissues? all of which you tell us are in complicated relation with the quantity and quality of the food which his system demands. What are the authorities to do in this difficult case? A question, as it appears to us, admitting but of one answer, embracing two particulars. 1. The allowance must be far above the minimum. 2. Such medical officers only should be appointed in whose judgment and general qualifications the strictest reliance can be placed, and upon these officers must devolve the duty and must rest the responsibility of apportioning the diet in every case, under certain limitations, and not in cases of sickness and infirmity only. There is also great reason in Mr. Hill's suggestion that *dif-*

*ferent* scales of diet should be adopted, and that prisoners, and we may add paupers, should be classified from time to time by the governor and surgeon according to the scale of diet which his system requires. Again, with his other qualifications, every surgeon of an union should be competent to determine the qualities and nutritive value of the food supplied, that is to say, to subject it with accuracy to chemical tests, and both to ultimate and proximate analysis. According to the present low scale of diet this is doubly necessary. If the object be to preserve health and to prolong life, these considerations are of the first importance. On the score of expense, these views may be said to be impracticable ; this in the face of facts cited and referred to in this article is lamentable. The variety and the abundance of food suited to the human constitution, produced by the land, and dispersed through the waters on the face of the globe, seem to forbid the idea to be entertained. Even Mr. Hill objects to the admission of luxury into prisons, (p. 423 ;) but in carrying out christian principles it cannot be avoided—a keen appetite, well satisfied, is the greatest of all luxuries. We must not enter upon this as a question of political economy, but the propriety of starvation as a punishment for three days (Class 1, p. 495) may fairly be doubted. Many of those who become amenable to this law are already half-starved wretches, who have never known three continuous days of good meals, at regular intervals, with moderate labour. It might on physiological grounds be better to feed a prisoner well, even of this class, and to trust to moral and religious influences to bring him to a sense of right and wrong. And as to the other classes, it may be fairly admitted, that in so far as a race of hardy felons and paupers, in perfect health, attaining a great age, is an incumbrance to the state, it would be an evil, but there still remains the question, why should they be an incumbrance?

X. On the Dietetical Regimen suited for Disordered States of the Digestive Organs. This is the last chapter of the work, and little more than a summary of some of the topics examined in former pages, as cookery, times of eating, the quantity of food taken at one meal, conduct before, at, and after eating, and the relative digestibility of different compound aliments; with exemplifications of the proper kind of food for dyspeptics. The following shall be our last quotation :

"Bulk is perhaps, nearly as necessary to the articles of diet as the nutrient principle. They should be so managed that one should be in proportion to the other. Too highly nutritive diet is, probably, as fatal to the prolongation of life and health as that which contains an insufficient quantity of nutriment. It has been ascertained that carnivorous animals will not live on highly concentrated food alone." (p. 525.)

The importance of food and diet as regards etiology has, we conceive, been well established by our previous quotations and comments. Interspersed in these volumes we find various illustrations of the practitioner's dependence upon it, not only in a prophylactic but in a remedial point of view. The use of water in the shape of slops, diluents, &c. to quench thirst, to augment the fluidity of the blood, to promote secretion, and probably "to promote the conversion of uric acid into urea" in fevers and acute inflammatory disorders ; of a *dry* diet to keep down the volume of the circulating organs, or to repress secretion, or to prevent thinness of the blood, as in valvular diseases of the heart, aneurism, &c., (Pereira, p. 84) for instance. To correct languor, hypochondriasis, and melancholia, and many of the complaints of the studious and sedentary, and of the idle and luxurious, Cheyne and Cadogan prescribed abstinence and labour. "Live upon sixpence a day and earn it," said John Abernethy. In the chapter on dietaries Dr. Pereira describes: 1, Full, common, or meat diet; 2, Animal diet; 3, Vegetable diet; 4, Spare or abstemious diet; 5, Fever diet; 6, Low diet; 7, Milk diet; 8, Dry diet; subjoining the Sick diet-tables of the metropolitan hospitals and other institutions. Each of these diets have their separate utility in the treatment of disease, which is very briefly described by the author. Besides these, we find mention made of several specific objects to be attained by means of aliment, as in the use of brown bread and oatmeal porridge in habitual constipation; of distilled water in the oxalate of lime diathesis; of cod-liver in rheumatic, gouty, and scrofulous diseases, and affections of the skin; of animal food and gluten bread in diabetes; and of lemon-juice and the potato, by virtue of their citric acid, as antiscorbutics. But on the dietetical treatment of the sick, the three volumes before us contain very little information, nor is there, as we conceive, a work extant, in which this highly important part of therapeutics has been properly or adequately treated of.

It has been the object of this article to show the extent, and to inculcate the importance of every part of the subject of diet, to all classes of the community, and as a fundamental department of his scientific inquiries, to the medical man in particular. We shall conclude with the remark, that it has a higher bearing than that which relates to the perfection of the physical frame of man. The moral and intellectual being is much more dependent upon it than the superficial observer is aware of; more, we will say, than the physical frame itself. The parent, of either sex, entails upon the offspring physical imperfections of constitution, by errors, excesses, or defects of diet and nutrition; the helpless being, from the earliest periods of its existence, being subject to painful where it ought to experience only pleasurable sensations. The infant at the breast receives a vitiated or an impoverished diet, unsuited to its digestive powers, or to the demands of the assimilating processes, inducing fretfulness and peevishness among the earliest complications of the affections then developing themselves. As childhood advances, the expanding mind becomes habituated to feelings of discontent, and the finer shades of mental character are superseded by others that naturally result from disappointment, continually flowing out of factitious and real wants unsupplied. The Sanatory Report to which we have before referred contains conclusive evidence, that a deteriorated physical condition is inimical to moral and intellectual cultivation. The teachers of the pauper children at Norwood aver, that the intellects of such children are torpid, it is difficult to gain their attention or to sustain it; they are irritable and bad tempered as compared with well-grown healthy children. A comparison was made between the progress of two sets of children in Glasgow, the one from the wynds, the other from a more healthy town district, and of a better physical condition. Although the former were placed under the best master, his pupils proved unable to keep pace with the better bodily conditioned children. Physical and mental aberration and degeneration proceed in parallel lines, till man finds himself an adult, the creature of artifice, and the slave of sensuality; or mentally and physically, the degenerate son of poverty.

Innumerable are the effects of evil habits of diet; of luxury in the rich, superfluity in the middling classes, want in the poor, and intemperance in all. Cheyne said of strong liquors, " besides producing diseases, they enrage the passions into quarrels, murder, and blasphemy. In those who have never known them, the sensations are more exquisite, the passions calmer, the appetites more controllable, and the health more uninterrupted." Dr. Pereira records that the temper of the leopard changes for the worse by being fed with two meals instead of one a day. We advocate not exclusive opinions. The temper of a gentleman of a sanguine temperament, who for some months lived upon vegetables, became much less excitable; and an individual of an opposite temperament was observed during the time he lived on a reduced diet to be more irritable. Irascible passions may undoubtedly be frequently subdued by attention to diet; and Galen was not presumptuous when he desired the teachers of philosophy to send for his treatment all persons of bad character. The intellectual faculties require for their due performance a proper supply of good blood to the cerebral organs. Instances might be quoted in which a diet of vegetables alone has appeared to be most conducive to the higher powers of the mind; instances also of the converse might be adduced. If we were taxed to enumerate those causes that counteract the beneficial influence upon society, of education, and the general diffusion of knowledge, we should class the errors committed by the community in reference to food, and the nutrition of the body, as the most prominent among the number.

The benefits that may accrue to mankind from the exertions, beyond all praise, of FATHER MATHEW, the apostle of *total abstinence* from the *most* seductive and pernicious of the inventions of luxury, it is impossible to divine. We may refer to the testimony of Mr. and Mrs. Hall in their work upon Ireland, to prove, that the humbler classes, are already gathering in a rich harvest of blessings from this regeneration. Individuals moving in higher circles, known to ourselves, who, objecting to the " pledge," have strictly followed out the principle, have also distanced evils with which they had been too long familiar. We have not yet heard of an instance attended with injurious consequences. The hygienic influence of the system adopted by Priessnitz is no mystery,—and an ample quantity of simple nutritious food, in the diet of Gräfenberg, rejecting every superfluity,—with the advantage of exercise in a fine air, amid delightful scenery,—have at least as much influence as *water*, in the cures that have been effected.

A most fallacious argument, adduced by the interested against moderation and temperance, requires a word of remark. Man, it is frequently urged, advances in years and attains a gray old age, without exercising any restraint upon his inclinations, and with no other guide but his natural appetites. Men who have been all their lives addicted to the use of large potations of spirituous fluids, are said to have reached seventy and even ninety years; and those who have partaken freely from their infancy of all the luxuries of the table, to have enjoyed an equally protracted existence. But the converse of the picture should be brought into view: the years cut short by gout, rheumatism, hepatic pulmonary and renal affections, heart diseases, imbecility, palsy, and apoplexy, and a host of effects leading to one certain goal, a premature decay both of the mental and physical being. Is the real period of man's probable existence, and the distinction between age and decrepitude ever considered? Instances of extreme longevity occur in all countries. In England, during the last century, several persons died more than 130 years old, and there are authentic records of many *hundreds* of individuals who have lived more than a century. The votaries of good living may think that an existence thus protracted is undesirable. Let them read Southwood Smith's Philosophy of Health: they will there find, admirably described, the distinction between advancing life, a period to which an indefinite number of years may be added, and decrepitude, which no human power can protract: and they will also find, eloquently displayed, the undoubted truth, that enjoyment is the only condition of life which is compatible with a protracted term of existence.

We have entered more fully than usual into the details of this subject, that we might carry conviction to the minds of our readers, of the importance which must attach to the possession of some definite principles, by which, as practitioners, we may be guided in laying down rules of diet. We cannot say that we regard Dr. Pereira's treatise either as complete or faultless. It is a compilation with an original arrangement of the matter. Many of Liebig's beautiful theories are introduced as extracts, without much and sometimes without any commentary; and notwithstanding objections raised against some of these, it is, as before intimated, essentially the application of the organic chemistry of Liebig and of the continental school, to the subject of diet. Many of our readers will regard the principles it comprises as too chemical, and we do not ourselves vouch for the stability of all the doctrines advanced, however cautiously, by the author. The numerous repetitions met with is a great fault, and the tables of analysis might have been concentrated with advantage, and placed together at the end of the work, or at the end of each section or chapter. Thus, the long table from Dr. Beaumont is first introduced piecemeal, and then again entire, under the head of "the digestibility of food." All this serves to increase the number of pages, or to exclude important matter, which otherwise, as we think, might have been introduced. The chemical annotation is also faulty. The chapter on alimentary principles might be curtailed with propriety, and a part of the matter transferred to that on compound aliments. Notwithstanding all this, the work is not only an excellent text-book to be placed in the hands of students, but one absolutely necessary for the use of every practitioner at the present moment, whether he has kept pace or not with organic chemistry in its recent rapid progression; it is calculated, by enabling the regular practitioner to found his directions for diet in some measure on a scientific basis, to prevent the abuse of the public mind by the empirical and frequently pernicious or ridiculous precepts of homœopathists, hydropathists, and charlatans. Dr. Pereira's original plan was to treat the subject in a more full and systematic manner; and we still think that a work embracing the principles of dietetics more extensively applied, the natural historical details more judiciously selected than we find them in Dr. Davidson's duodecimo, and the interesting facts so far as they are authentic, which Dr. Truman delights in, with their philosophical interpretation, and all that is important relating to the subject of food and diet, is still a desideratum in medical literature.

